# Predicting cell lineages using autoencoders and optimal transport

**DOI:** 10.1371/journal.pcbi.1007828

**Published:** 2020-04-28

**Authors:** Karren Dai Yang, Karthik Damodaran, Saradha Venkatachalapathy, Ali C. Soylemezoglu, G. V. Shivashankar, Caroline Uhler

**Affiliations:** 1 Institute for Data, Systems and Society, Massachusetts Institute of Technology, Cambridge, Massachusetts, United States of America; 2 Department of Electrical Engineering & Computer Science, Massachusetts Institute of Technology, Cambridge, Massachusetts, United States of America; 3 Department of Biological Engineering, Massachusetts Institute of Technology, Cambridge, Massachusetts, United States of America; 4 Mechanobiology Institute, National University of Singapore, Singapore; 5 FIRC Institute of Molecular Oncology (IFOM), Milan, Italy; 6 Department of Health Sciences and Technology, ETH Zurich and Paul Scherrer Institute, Villigen, Switzerland; 7 Department of Biosystems Science and Engineering, ETH Zurich, Zurich, Switzerland; Carnegie Mellon University, UNITED STATES

## Abstract

Lineage tracing involves the identification of all ancestors and descendants of a given cell, and is an important tool for studying biological processes such as development and disease progression. However, in many settings, controlled time-course experiments are not feasible, for example when working with tissue samples from patients. Here we present ImageAEOT, a computational pipeline based on autoencoders and optimal transport for predicting the lineages of cells using time-labeled datasets from different stages of a cellular process. Given a single-cell image from one of the stages, ImageAEOT generates an artificial lineage of this cell based on the population characteristics of the other stages. These lineages can be used to connect subpopulations of cells through the different stages and identify image-based features and biomarkers underlying the biological process. To validate our method, we apply ImageAEOT to a benchmark task based on nuclear and chromatin images during the activation of fibroblasts by tumor cells in engineered 3D tissues. We further validate ImageAEOT on chromatin images of various breast cancer cell lines and human tissue samples, thereby linking alterations in chromatin condensation patterns to different stages of tumor progression. Our results demonstrate the promise of computational methods based on autoencoding and optimal transport principles for lineage tracing in settings where existing experimental strategies cannot be used.

## Author summary

Many key biological processes, such as development and disease progression, require analyzing lineages of cells backwards as well as forwards in time. However, current single-cell experiments tend to be destructive to cells, so that a single lineage can only be measured at one point in time. In this work, we introduce a computational framework for predicting the lineage of cells from a single snapshot in time based on measurements of other cells at other time points. The method generates these lineages by computing the most plausible path for a population of cells to transition from one time point to the next, assuming that a cell is more likely to transition to similar cells compared to dissimilar cells. We validate our computational method on imaging data of fibroblasts and cancer cells, though our method could also be applied to other modalities of single-cell data such as genomics and transcriptomics as well as multi-modal single-cell datasets.

## Introduction

### Background

Lineage tracing during differentiation, development and disease progression is critical for studying the underlying biological mechanisms. Experimental strategies for lineage tracing include following cells over time using imaging or sequencing techniques after labelling them with synthetic or genetic markers [1–3]. However, many single-cell experimental methodologies are destructive to samples and thus only provide snapshots of these cellular processes in time and from different cells. This calls for computational approaches to lineage tracing that generate pseudo-lineages of cells based on single-cell datasets collected across different time points.

### Related work

A large number of strategies have been proposed in recent years for reconstructing pseudo-lineages of cells from single-cell transcriptomics data. These strategies (including Monocle [4], Monocle2 [5], SLICE [6], Waterfall [7], TSCAN [8], SCUBA [9], Wanderlust [10], Wishbone [11], PAGA [12], PBA [13] and others) generally combine techniques such as Principle Component Analysis (PCA), Independent Component Analysis (ICA), or t-Distributed Stochastic Neighbor Embedding (t-SNE) to project the data to a low-dimensional feature space with cluster-based, graph-based and/or curve-fitting methods for building the cell lineages. Overall, these strategies tend to make strong assumptions about the nature of the biological process (such as limited number of trajectories or branching points) and do not explicitly use time point information. Recently, Waddington-OT proposed to combine dimensionality-reduction with optimal transport to reconstruct pseudo-lineages from time-labeled gene expression data [14]. Unlike many previous methods, optimal transport does not make strong assumptions about the number of trajectories or branching points; instead it requires knowledge of discrete time labels in order to learn probabilistic couplings between cells that are sequential in time. Assuming that the time resolution of the data is high enough to capture sufficient representative cells from all of the transitional states, Waddington-OT presents a flexible and principled framework for reconstructing probabilistic pseudo-lineages of cells.

A key limitation of existing strategies for lineage tracing, including Waddington-OT, is that they rely on standard techniques such as PCA, ICA, or t-SNE to reduce the dimensionality of the data and generate a low-dimensional feature space in which to measure cell similarity [4–9]. Methods that use linear features given by PCA or ICA crucially rely on the assumption that linear distances between data samples in the measurement space accurately reflect cell similarity. However, this assumption is unrealistic in particular in high-dimensional measurement spaces, in which the data distribution may be supported on a complex low-dimensional manifold. Other dimensionality-reduction methods such as t-SNE are non-linear but irreversible and do not enable interpretation of the feature space. Strategies that use nearest-neighbor distances other than Euclidean distances to measure cell similarity aim to take the data manifold into account [10–13]. However, these distances are still susceptible in high-dimensional spaces, and more crucially, directions and trajectories along the cell manifold are not easily interpreted with respect to the original measurement space. With the advent of many new technologies for collecting various types of high-dimensional single-cell data, there is a growing need for computational strategies that learn interpretable feature spaces that capture meaningful semantic relationships between cells, using one or even multiple single-cell modalities. Advances in machine learning in the past decade have prompted new computational methods for learning from high-dimensional data such as images [15–17], but these are primarily aimed at the classification of cells and tissues [18–20] and also do not offer interpretable features for key downstream tasks such as the computational reconstruction of cellular pseudo-lineages.

### Contributions

**Fig 1:**
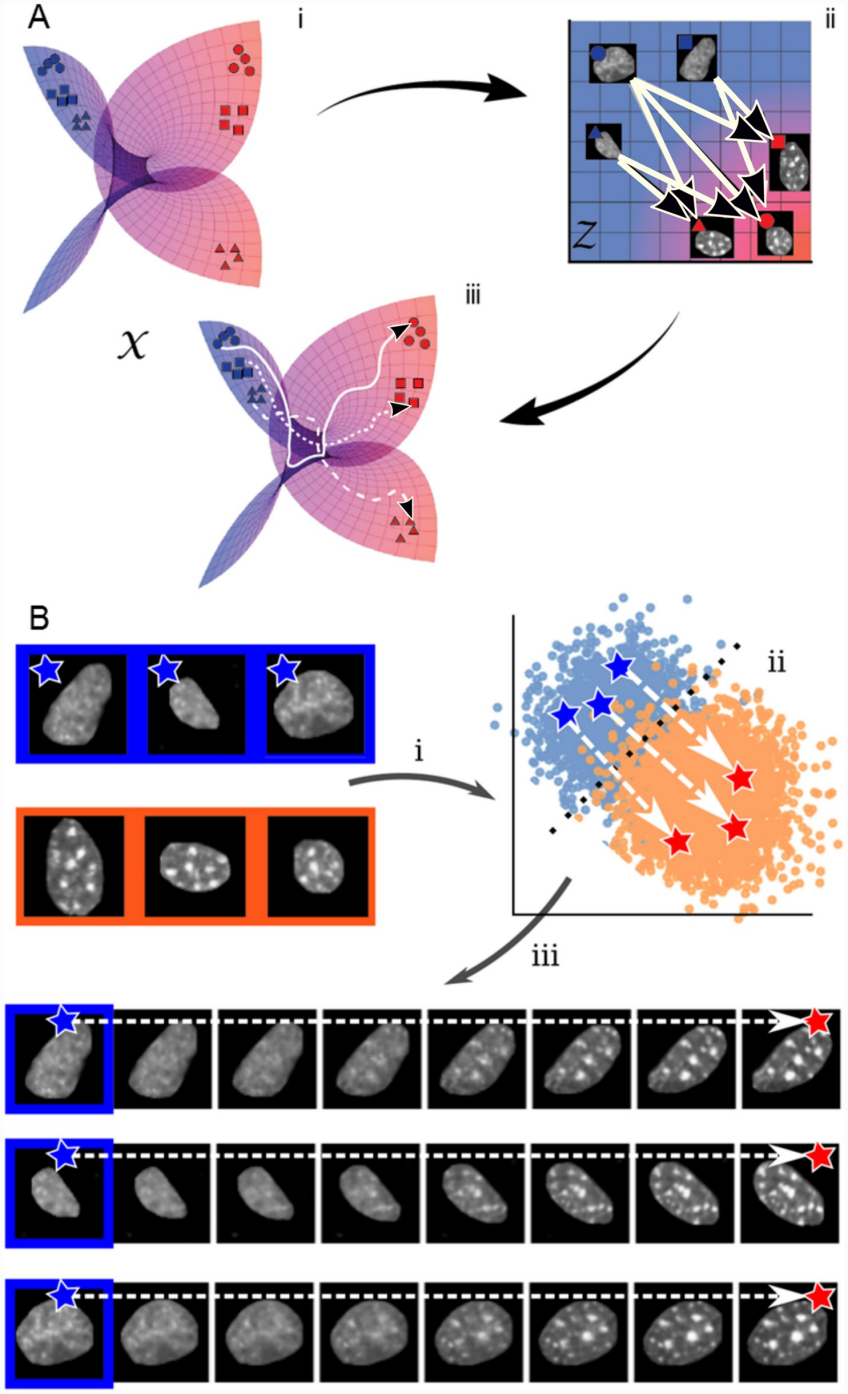
Schematic of ImageAEOT for tracing cell trajectories. (A) X represents the manifold of images taken from a population of cells during the biological process of interest. The objective is to trace a trajectory between two single-cell datasets (e.g. blue and red), taken from two distinct time points of interest. First, the images are mapped to a feature space Z using an autoencoder. In the feature space, optimal transport methods are used to trace trajectories from the two single-cell datasets. The arrows indicate probabilistic trajectories predicted by optimal transport. Finally, using the autoencoder, the feature space trajectories are mapped back to the image space, which can be visualized as smooth trajectories from an image from one dataset to its matched image from the other dataset. (B) Illustration of ImageAEOT using nuclear images of MCF7 cells (source population) and NIH3T3 cells (target population). The end points of the predicted trajectories are generated images, i.e. ImageAEOT does not merely interpolate between two given images but rather generate nuclei that have the features of nuclei in the target population, but still resemble the given cell nucleus in the source population.

In this paper, we present a novel strategy for reconstructing pseudo-lineages from high-dimensional single-cell datasets—ImageAEOT—which builds on Waddington-OT by using an autoencoder to embed single-cell data into an interpretable feature space in order to perform optimal transport. An overview of the method is shown in Fig 1a. First, we embed the datasets into a low-dimensional feature space using an autoencoder [21–23] that is designed to capture semantically meaningful features. Within the feature space, we learn a probabilistic coupling between cells based on the principles of optimal transport theory [24–27] and trace linear trajectories between matched cells. To reconstruct the pseudo-lineages and interpret the functional features and biomarkers that are changing along these trajectories, we decode the cell features back to the original data space. Overall, given a cell measurement from one stage of a biological process, our method can be used to reconstruct a pseudo-lineage of this cell that retains its original characteristics and also captures the population features of earlier and later stages (Fig 1b). To benchmark the accuracy of our approach against existing methods, we introduce a new computational lineage-tracing task: reconstructing pseudo-lineages of cells from single-cell imaging datasets of a fibroblast (NIH3T3) and cancer (MCF7) *in vitro* co-culture system. Finally, we provide qualitative examples over multiple single-cell imaging datasets to show that our model enables the interpretation of functional features and biomarkers underlying a biological process. While the evaluation in this work focuses on single-cell imaging datasets, our method is broadly applicable to other modalities of single-cell data (e.g., genomics and transcriptomics data) as well as multi-modal single-cell datasets (e.g., genomics data combined with imaging data).

## Methods

### Model

#### Statement of problem

Given two single-cell datasets (e.g., collected at two time points), lineage tracing can be formulated as the problem of finding a probabilistic coupling between the two datasets that indicates the probability that each pair of cells belongs to the same lineage [14]. Even though the two single-cell datasets may not actually contain any common lineages, since in practice they may be collected from different cell samples, the idea is to match cells from one dataset with cells from the other dataset that are representative of their ancestors or descendants. This process can be repeated for multiple input single-cell datasets to reconstruct pseudo-lineages over many time points. Concretely, given *M* and *N* cell samples from the two datasets respectively, we seek an *M* × *N* probability matrix *P* = (*p*_*ij*_) satisfying ∑_*k*_ *p*_*ik*_ = 1 for all *i* ∈ [*M*], where we used the conventional notation [*M*] = {1, …, *M*}. Here, the matrix entry *p*_*ij*_ represents the probability that cell *i* from the first dataset belongs to the same lineage as cell *j* from the second dataset.

#### Proposed approach

The above problem can be formulated as a discrete optimal transport problem [14] and solved using matrix scaling algorithms for optimal transport [27]. Optimal transport (OT) is a framework for comparing two distributions by finding a way to “push” or re-distribute one distribution to the other while incurring minimal transport cost. Formally, given the vectors *a* = **1**_**M**_/*M* and *b* = **1**_**N**_/*N* representing uniform discrete probability distributions over the *M* and *N* samples from the two respective datasets, Kantorovich OT seeks an *M* × *N* matrix $\tilde{P}=({\tilde{p}}_{ij})$ minimizing

$$\begin{array}{c} \min_{\tilde{P}}\sum_{i,j}c\left(i,j\right){\tilde{p}}_{ij} \end{array}$$ (1)

subject to $\tilde{P}1_{N}=a$ and ${\tilde{P}}^{T}1_{M}=b$, where *c*(*i*, *j*) denotes the cost of matching cell *i* from the first dataset to cell *j* from the second dataset. For moderate-sized datasets, this matrix can be solved for efficiently using the Sinkhorn algorithm for regularized OT [27]. An appropriate scaling of $\tilde{P}$ (by *M* if reconstructing lineages forwards in time, and by *N* if reconstructing backwards in time) then yields the desired probability matrix *P* given in the problem statement.

The solution to Eq (1) depends heavily on the cost function *c* for matching samples from the two datasets. For single-cell gene expression datasets, Euclidean distance between samples *i* ∈ [*M*] and *j* ∈ [*N*], either measured in the original gene expression space or in a feature space obtained by PCA, has been used as the cost *c*(*i*, *j*) [14]. The accuracy of the pseudo-lineages constructed using this cost function relies on the crucial assumption that straight-line distances between data samples accurately reflect lineal relationships between cells. However, when working with extremely high-dimensional single-cell data, it is unlikely that this cost function is appropriate.

Instead of using linear distance between cells as a measure of cell similarity, we propose using a cost function that is *directly learned from the data using an autoencoder*. Specifically, let **X** = (*X*^(1)^, *X*^(2)^) be the combined datasets of samples from the measurement space X and let $Z=R^{d}$ be a lower-dimensional feature space. In practice, we used *d* = 128 throughout this work. In addition, while in this work X represents single-cell imaging data, it can represent the measurement space of any single-cell data modality or combinations thereof. The feature space Z is learned by jointly training an *encoder function* E:X→Z and a *decoder function* D:Z→X, both parameterized using neural networks, to minimize the loss function

$$\begin{array}{c} \min_{E,D}E_{x∼X}||D\left(E\left(x\right)\right)-{x||}_{2}^{2}+\ell \left(X;E,D\right), \end{array}$$ (2)

where the first term is the standard dataset reconstruction error for autoencoders and the second term represents an additional loss function or regularization of the loss function (discussed in Eqs (3) and (4) below). Once the encoder and decoder functions are learned, we compute a probabilistic matching between the two single-cell datasets *X*^(1)^ and *X*^(2)^ using optimal transport as described above using the cost function $c\left(i,j\right)=||E\left(X_{i}^{\left(1\right)}\right)-E\left(X_{j}^{\left(2\right)}\right)\left|\right|$, i.e., the linear distance between cells in the feature space learned by the autoencoder. Equivalently, this strategy can be thought of as first embedding the data into the feature space using the encoder, performing optimal transport in the feature space, and then mapping back to the measurement space using the decoder.

This approach has two key advantages as compared to existing methods such as Waddington-OT [14]. First, the linear distance between cells in the feature space translates to a non-linear distance in the measurement space that may better reflect semantic relationships between cells. Second, the decoder function enables the interpretation of the functional features and biomarkers associated with the pseudo-lineages, namely by decoding these from the feature space back to the measurement space. In particular, we propose to facilitate model interpretation by *interpolating* the predicted pseudo-lineages and decoding them to the measurement space by computing the following function

$$\begin{array}{c} T_{z,\tilde{z}}\left(t\right)=D(\left(1-t\right)*z+t*\tilde{z}), \end{array}$$

where *D* is the learned decoder function, $z,\tilde{z}$ are the matched cells to be interpolated in the feature space and *t* ∈ [0, 1] is a fractional value that represents a point along the straight-line path between *z* and $\tilde{z}$. The main purpose of the interpolation and decoding is to provide qualitative understanding and validation of distances in the feature space. Additionally, when the measured datasets are close enough in time (such that intermediate time points would fall on a line in the feature space), the interpolated pseudo-lineages can also be interpreted as a prediction of intermediate stages between these time points.

As described in Eq (2), the feature space and generative quality of the autoencoder can be improved by adding terms other than the reconstruction loss to the loss function. For example when studying differentiation, cell type data may be available for later time points and the objective may be to reconstruct pseudo-lineages to predict the cell fate decisions made by cells from earlier time points. The feature space in such problems should ideally capture the differences between cell types in later time points. In such problems, we propose to add a weighted classification term to the standard reconstruction loss: Given a vector of class labels Y∈Y with the same cell index as **X** (which may contain empty entries if the cell type is not available), and a classifier function *f*, the optimization problem then becomes

$$\begin{array}{c} \min_{E,D}E_{x∼X}||D\left(E\left(x\right)\right)-{x||}_{2}^{2}-\lambda E_{\left(x,y)∼(X,Y\right)}\sum_{\tilde{y}\in Y}1_{y=\tilde{y}}\text{log}\;f_{\tilde{y}}\left(E\left(x\right)\right), \end{array}$$ (3)

where λ > 0 is a hyperparameter. In addition, to improve the quality of the generated images of the autoencoder (e.g., for interpretability of pseudo-lineages), we propose to augment the loss function by a regularizator, a key ingredient of current generative autoencoder models (e.g., variational autoencoders [23], adversarial autoencoders [28], Wasserstein autoencoders [29], etc.). The intuition behind such regularization terms is to force the data distribution in the feature space to be more similar to an isotropic Gaussian distribution. As proof-of-concept, we implemented variational autoencoders in this work, but we emphasize that any other generative autoencoder model could be used. In particular, we used a probabilistic rather than deterministic encoder *E* and added a KL-divergence term *D*_*KL*_ to the autoencoder loss function, namely

$$\begin{array}{c} \min_{E,D}E_{x∼X}||D\left(E\left(x\right)\right)-{x||}_{2}^{2}+\lambda D_{KL}\left(p_{E}\left(\cdot |x\right)|p_{g}\left(\cdot \right)\right), \end{array}$$ (4)

where λ > 0 is a hyperparameter, *p*_*g*_(⋅) is an isotropic Gaussian distribution and *p*_*E*_(⋅|*x*) is the feature distribution of sample *x* under the probabilistic encoder *E*. While our method can be applied to arbitrary modalities of single-cell data (e.g., genomics or transcriptomics data) as well as multi-modal single-cell datasets (e.g., genomics data combined with imaging data), the evaluation in this work focuses on single-cell imaging datasets. As a result, we chose a convolutional architecture for our neural network as shown in S2 Fig.

#### Implementation

Neural network models were implemented in Python using the Pytorch library [47] and trained on an NVIDIA GeForce GTX 1080TI graphics card. The architecture of the VAE model in the ImageAEOT is shown in S2 Fig. Hyperparameter values of 0, 1e-6, 1e-7, 1e-8 were used in the objective. This model was trained using the Adam optimizer, a popular variant of stochastic gradient descent [48], with learning rate initialized at 1e-4, and batch sizes of 64 images. For classification tasks in the latent space, we implemented either a linear network or a feedforward network with ReLU activations and two hidden layers. The models were trained using the Adam optimizer with learning rate initialized at 1e-3. The VoxNet model architecture [49] used for classifying whole images is shown in S2 Fig. For performing latent discriminant analysis in our pipeline, we used the Python scikit-learn library [46]. For the feature ablation studies, feature extraction was done using the Python mahotas library [34], and the logistic regression model was implemented using scikit-learn [46].

### Cell-culture experiments

#### Reagents used

NIH3T3 (CRL-1658), BJ (CRL-2522), MCF10A (CRL-10317), MCF7 (HTB22) and MDA-MB-231 (HTB-26) cells were obtained from ATCC. They were cultured in DMEM-high glucose (ThermoFisher Scientific 11965092) media supplemented with 10%FBS (ThermoFisher Scientific 16000044) and 1% pen-strep (Sigma P4333) antibiotic. Antibodies used: EpCAM (Cell Signaling Technology, 2929S) and Vimentin (Cell Signaling Technology, 5741S). Other reagents: Breast tissue sections within normal limits (CS708873, Origene), metastatic breast adenocarcinoma tissue sections (CS548359, Origene), Histozyme (H3292-15ML, Sigma), ProLong^®^ Gold Antifade Mountant with DAPI (P36941, ThermoFischer Scientific), Paraformaldehyde PFA (Sigma, 252549-500ml), Triton (Sigma, X100-100ml) and DAPI solution (ThermoFisher Scientific R37606) and IF wash buffer (for 250ml: 125mg NaN3 + 500*μ*l Triton X-100 + 500*μ*l Tween-20 in 1X PBS).

#### Micro-contact printing

Fibronectin micropatterning was performed as described by Makhija et al [50]. Briefly, circular fibronectin (Sigma F1141-2MG) micropatterns (area = 1800*μ*m2) were made on uncoated Ibidi dishes (81151). These micropatterned dishes were then passivated with 0.2% pluronic acid (Sigma P2443) for 10 minutes and washed with PBS.

#### Co-culture experiment

Cell culture and experiments were all performed at 37°C, 5% CO2 and humid conditions. MCF7 cells were seeded previous day (Day -1) on 1800 *μ*m2 circular fibronectin micropatterns. After 24 hours, MCF7 clusters (cluster of 10 cells) were obtained. Collagen gel (1mg/ml) was prepared as per the manufacturer’s instructions along with DMEM media. MCF7 clusters were then scraped off and mixed with 30,000 NIH3T3 fibroblasts in 300*μ*l of collagen gel solution which was then added to an uncoated Ibidi dish. For control conditions, either only MCF7 clusters or 30,000 NIH3T3 cells were added with 300*μ*l of collagen gel solution in each Ibidi dish. The gel was then allowed to solidify at 37°C. After two hours, 500*μ*l of fresh media was added to these samples. Each set of dishes were then fixed on Day1, Day2, Day3 and Day4.

#### IF staining

For IF staining, media was aspirated and 4%PFA was added and incubated for 20 minutes. The samples were then washed thrice with PBS + glycine buffer. The gel was treated with 0.5% Triton for 20 minutes to permeabilize the cells followed by washes with the PBS + glycine buffer. The samples were then blocked with 10% goat serum in IF wash buffer (blocking solution) for 2 hours. Primary antibodies in blocking solution were then incubated overnight as per the dilution recommended by the manufacturer. Next day, the samples were washed thrice with IF wash buffer for 5 minutes each. Secondary antibody in blocking solution was then added as per the manufacturer’s instruction for 2 hours. Samples were then washed thrice with IF wash buffer for 5 minutes each. DAPI solution was added to the samples and stored temporarily at 4°C until imaged.

#### Immunohistochemistry

Formalin-fixed, paraffin-embedded (FFPE) tissue sections (5μm thickness) on slides were deparaffinized by heating them in an oven at 60°C for 5 minutes and subsequently washing them with xylene. The sections were then rehydrated in serially diluted ethanol solutions (100%—50%) as per standard protocols and rinsed with water. Antigen retrieval was performed using Histozyme solution as per the manufacturer’s protocol and then rinsed with water. DAPI was then added to these sections and they were covered with a coverslip. The slides were incubated for 24 hours after which the coverslips were sealed and taken for imaging.

#### Imaging

Most of the solution in the dish was aspirated before imaging. Around 50*μ*l of the solution was left to prevent drying of the collagen gel. The images were obtained using a Nikon A1R confocal microscope. For co-culture samples, Z-stack images were captured using 40X objective (water, 1.25 NA), 0.3*μm* pixel, Z-depth of 1*μm* and all images were captured for not more than 50*μm* depth. Each image is 1024X1024 pixels in size. For larger field images, 2 × 2 images or 3 × 3 images were obtained and stitched together with 5% overlap. For tissue slices, wide-field images were obtained using an Applied Precision DeltaVision Core microscope with 100X objective (oil, NA 1.4) and a pixel size of 0.2150*μm*. These 512 × 512 12-bit images were deconvolved (enhanced ratio, 10 cycles) and saved in .tiff format.

#### Segmentation of nuclei

Images were analyzed using custom codes written in ImageJ2/Fiji [51]. The raw 3D images labelled for DNA using DAPI, acquired using a laser scanning confocal microscope, were filtered using a Gaussian blur and thresholded using automated global thresholding method such as Moments to binarize and identify nuclear regions. Watershed was used to separate touching nuclear regions. This binary image was then used to identify individual nuclei as 3D objects within a size range of 200-1300*μm*^3^. Each nucleus identified as a separate 3D object was visualized with distinct colors. In order to smoothen any irregular boundaries, a 3D convex hull was carried out and then the individual nuclei were cropped along their bounding rectangles and stored. This was carried out using the functions from Bioformats and the mcib3d library. In order to separate nuclei that were clumped and could not be separated using watershed, the 3D Euclidian distance transform was carried out on these clumps of nuclei followed by a second round of thresholding to remove pixels from the boundaries; then individual nuclei were identified as described earlier. From this set, the blurred out of focus nuclei and the over-exposed nuclei were filtered out and then the selected nuclei were used for further analysis.

## Results

### Benchmark task for computational lineage tracing

**Fig 2:**
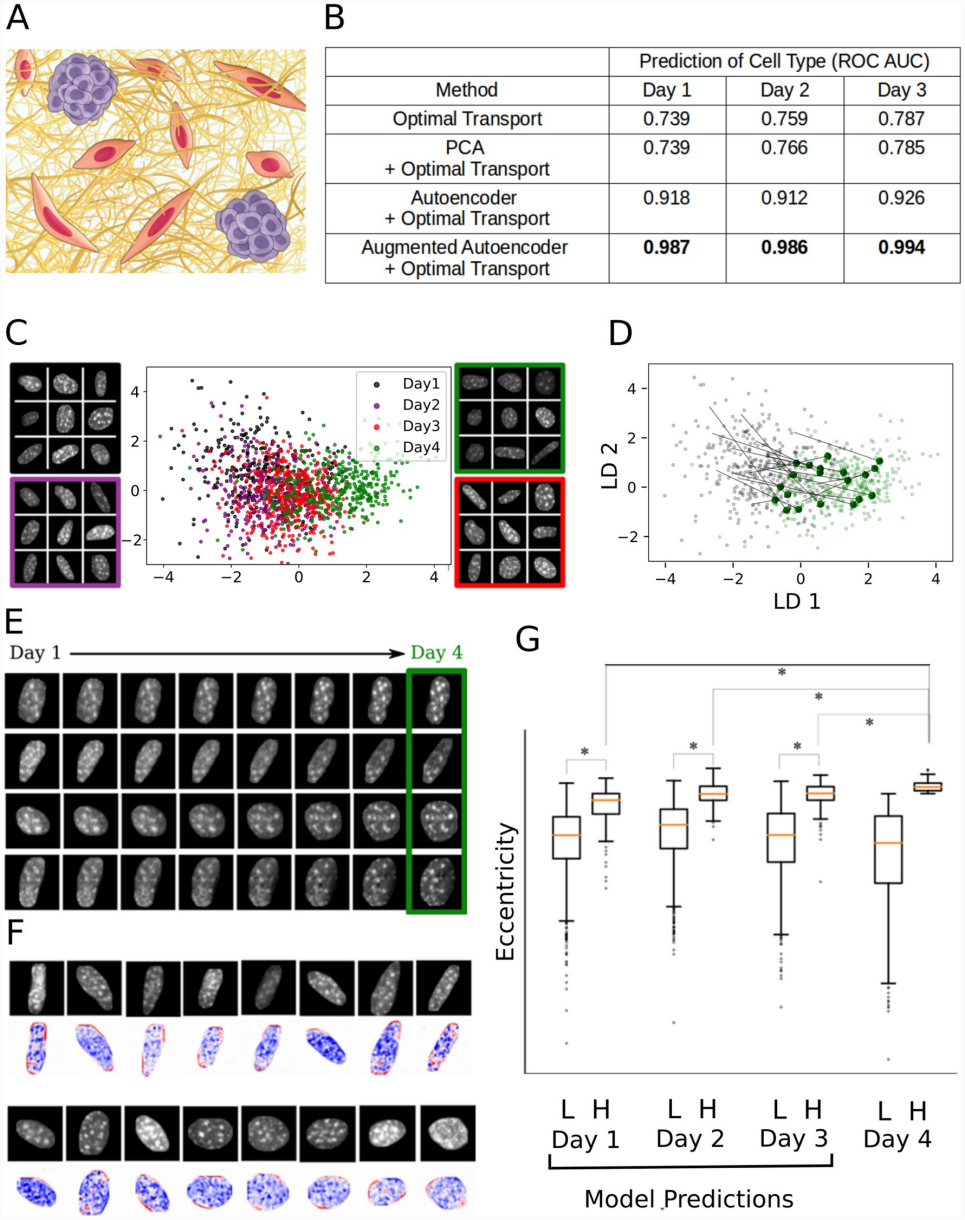
ImageAEOT applied to lineage tracing of single-cell imaging data from a fibroblast-tumor coculture system. (A) Schematic of the fibroblast-tumor coculture experiment. NIH3T3 fibroblasts were cocultured with MCF7 cells for 1 to 4 days. The cells were subsequently fixed, DAPI stained, and imaged. (B) Comparison of ImageAEOT using a standard autoencoder (AE+OT) and an augmented autoencoder (augAE+OT) against Waddington-OT (OT, PCA+OT) on the proposed benchmark task. The evaluation metric shown is the area under the receiver operating characteristic curve (ROC-AUC) of the inferred probabilistic labels (score between 0 and 1, higher is better). ImageAEOT achieves considerable quantitative gains over the baseline. (C) Visualization of NIH3T3 nuclei from Days 1-4 in both the original image space and the feature space using a Linear Discriminant Analysis (LDA) plot. The x-axis and y-axis are respectively the first and second linear discriminants applied to the data. The first two linear discriminants capture the smooth progression of the cells from Day 1 through Day 4. Day 1: black; Day 2: purple; Day 3: red; Day 4: green. (D) Predicted trajectories in the feature space using optimal transport, plotted on the same set of axes as in (C). ImageAEOT was used to trace the trajectories of Day 4 NIH3T3 nuclei back to Day 1 NIH3T3 nuclei. Each black arrow is an example of such a trajectory. (E) Predicted trajectories mapped back to the image space. Note that only the last image in each sequence is a real Day 4 NIH3T3 nucleus. All other images are predicted and generated by ImageAEOT. (F) Interpretation of feature space by perturbing cell features and decoding the results to the image space. The top row shows nuclear images from Day 1 NIH3T3 fibroblasts. The image shown below each top row image is the predicted change in the image if we were to shift it towards the distribution of Day 4 images in the feature space, along the first linear discriminant shown in (C) (blue: decrease in pixel intensity; red: increase in pixel intensity). These results suggest that the elongated nuclei become more elongated (i.e. increase of intensity at the poles) and the more spherical nuclei remain more spherical as the fibroblasts progress from Day 1 to Day 4 during their activation. In addition, fibroblast activation is accompanied by chromatin decondensation as revealed by the decrease in pixel intensities. (G) NIH3T3 nuclei were divided into two sub-populations on Day 4 based on nuclear elongation (L: low eccentricity, H: high eccentricity) and ImageAEOT was used to generate pseudo-lineages of these sub-populations on Days 1-3. The box-plots show the distribution of elongation of the Day 4 cells as well as model predictions on Days 1-3. The model predictions suggest that the elongated sub-population on Day 4 is already detectable on Day 1, i.e., a subset of the Day 1 population is already primed for activation.

Reconstructed pseudo-lineages are generally challenging to evaluate due to the absence of ground truth lineages. Validation for lineage tracing based on gene expression data has been proposed using genetic tracers [30]. To evaluate ImageAEOT, we propose a novel benchmark task for computational lineage tracing using single-cell imaging data. The dataset consists of 6479 chromatin images taken over multiple time points of fibroblasts (NIH3T3) and tumor cells (MCF7) embedded in 3D engineered tissues (Fig 2a and S1 Fig). This mimics their interaction in the tumor microenvironment [31–33]. In addition, we provide ground-truth binary labels for the cell type, obtained by measuring the relative levels of Vimentin (enriched in NIH3T3 cells) and Epcam (enriched in MCF7 cells) via immunofluoresence staining. The benchmark task involves reconstructing pseudo-lineages using the chromatin images from Days 1-4 and cell type labels from Days 1-3 and evaluating these pseudo-lineages to infer the labels of cells from Day 4. We assessed the performance of several methods: (1) optimal transport on original measurements; (2) optimal transport on PCA features; (3) ImageAEOT using a standard autoencoder, and (4) ImageAEOT using an augmented autoencoder (Fig 2b). Methods (1-2) are existing baseline methods based on optimal transport that use linear features [14], while ImageAEOT (3-4) uses learned features of an autoencoder. For the evaluation metric, we compute the area under the receiver operating characteristic (ROC) curve from the inferred label probabilities. Overall, we found that ImageAEOT outperforms the baselines, demonstrating the quantitative gains of ImageAEOT over applying optimal transport directly to cell measurements or to standard linear features. We performed similar benchmark analysis using cell morphology features [34] as the labels and also found that our strategy outperforms the baselines (S11 Fig).

### Application of ImageAEOT to fibroblast activation

Having shown the quantitative improvements of ImageAEOT features over standard features, we now apply our method towards reconstructing and visualizing pseudo-lineages in various biological systems. Importantly, an advantage of ImageAEOT is that the autoencoder can map cell features back to the measurement space, which facilitates interpretability of the model predictions. First, we apply ImageAEOT towards visualizing the fibroblast pseudo-lineages generated by ImageAEOT using the NIH3T3 images from our benchmark dataset. By Day 4, a subset of fibroblasts are activated and ImageAEOT infers the state of these cells in earlier time points. Fig 2c shows the low-dimensional latent representation of the NIH3T3 nuclear images obtained by the autoencoder. The model was tuned to ensure that images can be encoded and decoded with high fidelity, while also maintaining the geometry of the low-dimensional image manifold in the latent space (S3 Fig). As a consequence, classifying cell states using deep convolutional neural networks based on their latent representation achieves a comparable level of accuracy as when classifying cells based on the original images (S4 and S6 Figs). We then back-traced NIH3T3 cells from Day 4 to Day 1 in the latent space (Fig 2d and S12 Fig) and generated the corresponding single-cell image trajectories using ImageAEOT (Fig 2e). Note that only the last image in each sequence is real; the others are generated by decoding averaged pseudo-lineages from the feature space to the measurement space and facilitate the interpretation of the optimal transport model. These examples highlight a key advantage of ImageAEOT—the ability to interpret the model predictions by mapping cell features back to the measurement space. They also provide qualitative validation of the feature space for optimal transport; note that the interpolations between cells in the feature space lie on the cell manifold, which suggests that straight-line distances in the feature space are meaningful for measuring cell similarity compared to linear distance in the measurement space.

The autoencoder component of ImageAEOT also enables interpretation of different directions in the feature space, namely by adding small perturbations to cell features and comparing the corresponding single-cell images in the original measurement space. This can be used to identify biomarkers of the fibroblast activation process by adding a perturbation along the linear discriminant function that distinguishes Day 1 from Day 4 fibroblasts. As shown in Fig 2f, the fibroblast activation process involves an increase in nuclear elongation and alterations in chromatin condensation patterns. Importantly, this analysis reveals that fibroblast subpopulations that are more elongated in Day 1 become more activated in Day 4 (Fig 2g), suggesting that ImageAEOT can identify subpopulations of fibroblasts in the heterogeneous tissue microenvironment that are primed for activation. This aligns well with known biology of fibroblast activation, where recent studies have shown that the activation process is accompanied by increased cytoskeletal to nuclear signaling, increased nuclear elongation, and alterations in chromatin condensation patterns to facilitate activation-related gene expression programs [31–33, 35]. A similar analysis was performed on the MCF7 nuclei during the activation process (S3, S5 and S6 Figs).

### Application of ImageAEOT to breast cancer cell lines and tissues

**Fig 3:**
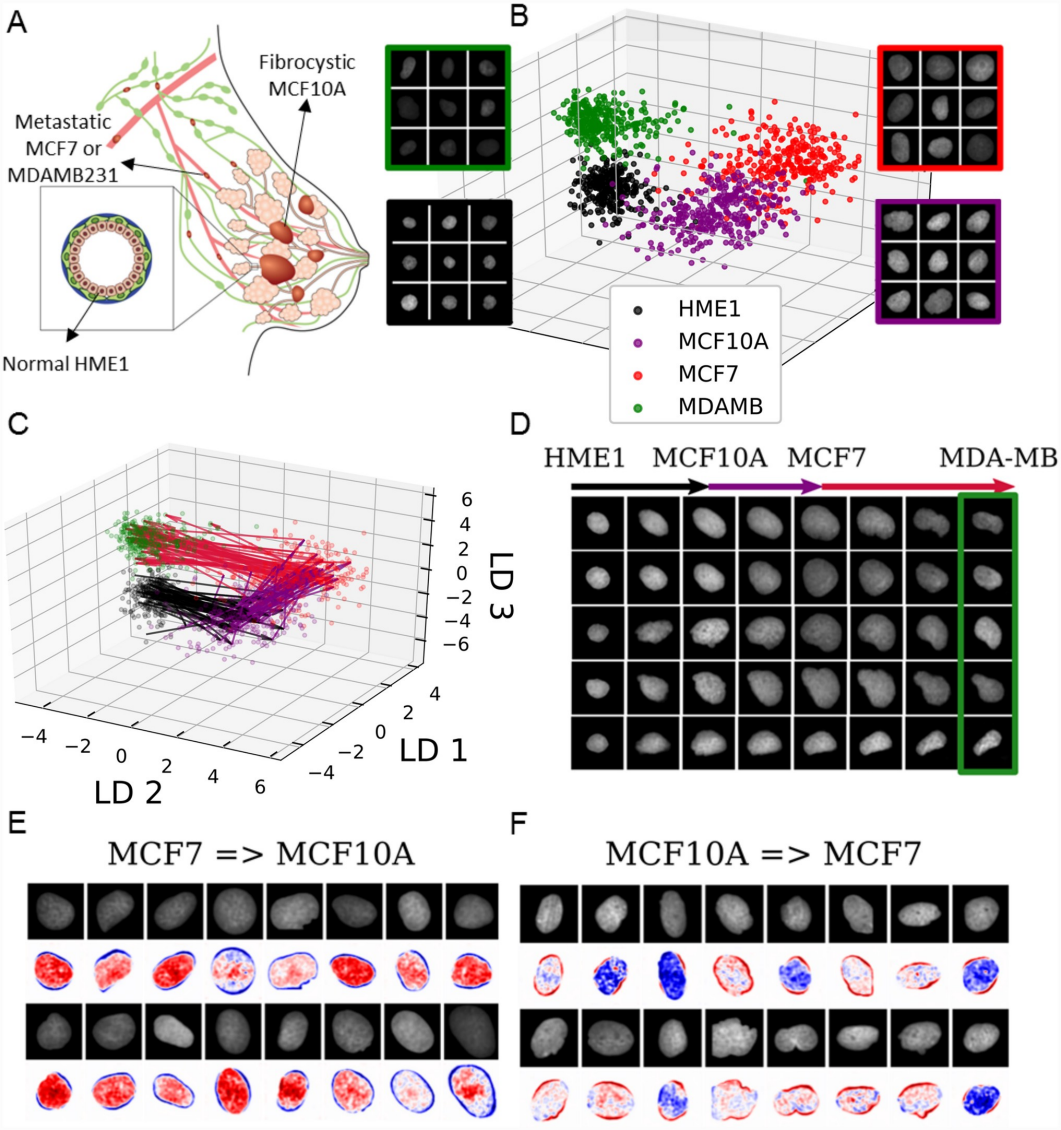
ImageAEOT applied to tracing cellular trajectories during breast cancer progression. (A) Schematic of breast cancer progression. Normal epithelial cells (HME-1) in the breast may become fibrocystic (MCF10A), develop into cancer cells (MCF7), and finally become highly invasive (MDA-MB231). (B) Visualization of nuclear images from four breast cell lines in both the original image space and the feature space using a Linear Discriminant Analysis (LDA) plot. The x-axis, y-axis, and z-axis are respectively the first, second and third linear discriminants applied to the data. HME-1: black; MCF10A: purple; MCF7: red; MDA-MB231: green. (C) Predicted trajectories in the feature space using optimal transport, shown on the same axes as in (B). ImageAEOT was used to trace the trajectories from HME-1 to MCF10A to MCF7 to MDA-MB231. (D) Predicted trajectories mapped back to the image space. Note that only the final image in each sequence is a real MDA-MB nucleus; the remaining images are predicted and generated by ImageAEOT. (E-F) Illustration of the principal features that change between MCF10A and MCF7, namely a combination of nuclear morphological and chromatin condensation features.

Next, we applied ImageAEOT to model and visualize trajectories of nuclei progressing through various stages of breast cancer (Fig 3a). The dataset consists of 1284 nuclear images of HME-1 (normal breast epithelial) cells, MCF10A (fibrocystic epithelial) cells, MCF7 (metastatic breast cancer) cells, and MDA-MB231 (highly invasive metastatic breast cancer) cells. As described in the earlier sections, the first step of ImageAEOT involves learning a latent representation of the nuclear images that captures their low-dimensional structure (Fig 3b). The quality of the autoencoder is evidenced by accurate reconstruction, sampling, and classification results (S7 and S8 Figs). ImageAEOT was then used to back-trace the trajectories of nuclei from the highly invasive state (MDA-MB231) to normal breast epithelial state (HME-1) in the latent space (Fig 3c). Decoded to the image space, these trajectories yield predictions of how normal mammary epithelial HME-1 cell nuclei may progress through fibrocystic or metastatic stages of breast cancer (Fig 3d). We also analyzed the principal nuclear features that change between the various cell types by adding small perturbations to the latent representations and decoding them to the image space. This analysis shows that the principal features of the transition between MCF10A and MCF7 involve both alterations in nuclear morphology and chromatin condensation patterns (Fig 3e and 3f), while the transition between other pairs of cell types (HME-1-to-MCF10A and MCF7-to- MDA-MB231) are mainly characterized by nuclear morphological changes (S9 Fig).

**Fig 4:**
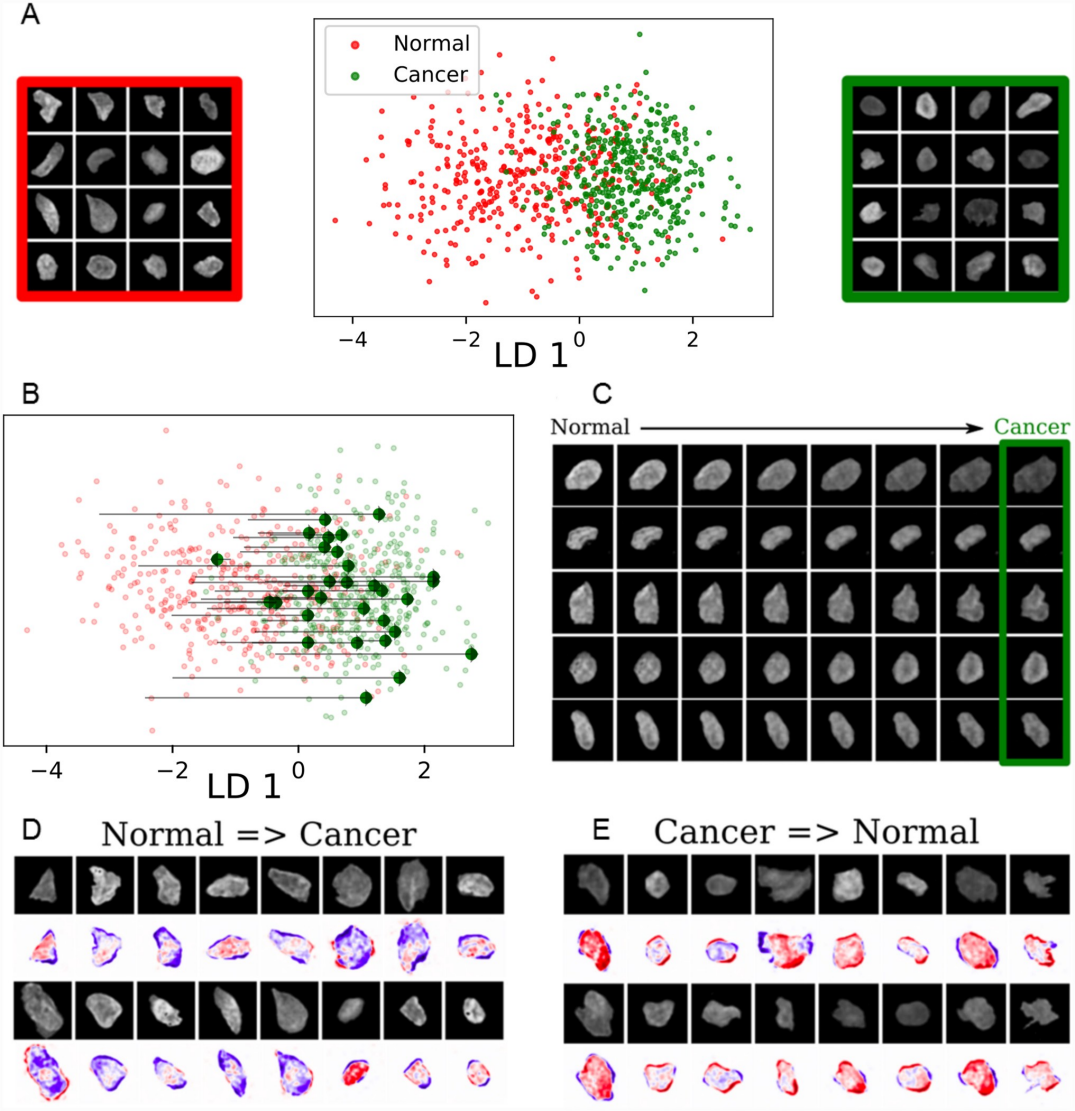
ImageAEOT applied to tracing cellular trajectories in breast tissues. (A) Visualization of normal and cancer nuclear images from tissue in both the original image space and the feature space using a Linear Discriminant Analysis (LDA) plot. The x-axis shows the first linear discriminant applied to the data. (B) Predicted trajectories in the feature space using optimal transport, shown on the same axes as in (A). ImageAEOT was used to trace the trajectories from normal to cancer nuclei. (C) Predicted trajectories mapped back to the image space. Note that only the last image in each sequence is a real nucleus; the remaining images are predicted and generated by ImageAEOT. (D-E) Illustration of the principal features that change between normal and cancer nuclei, namely a combination of nuclear morphological and chromatin condensation features.

Finally, we applied ImageAEOT to model trajectories of nuclei in human breast tissues. The dataset consists of 840 nuclear images from normal breast epithelial tissue as well as breast cancer tissue. ImageAEOT learns a high-quality latent representation of these images (Fig 4a), indicated by our reconstruction and sampling results (S10 Fig). The trajectories of nuclei from normal to cancerous cells in the latent space are shown in Fig 4b and the decoded images in Fig 4c. Within the tissue microenvironment the principal features corresponding to the transition between normal and cancer cells involve both alterations in nuclear morphology and chromatin condensation patterns (Fig 4d and 4e). Our results are consistent with known biology of tumor progression, where alterations in nuclear morphological and chromatin condensation patterns have been shown to go hand-in-hand with the onset and progression of cancer [36]. Collectively, these results suggest that ImageAEOT can be applied to identify early physical biomarkers of cancer initiation and progression.

## Discussion

Understanding cell-state transitions during development or disease progression is a major challenge in modern biology and depends on lineage tracing [37–39]. However, in many settings, controlled time-course experiments are simply not feasible—for example, when working with tissue samples from patients—making it a challenge to model single-cell trajectories. ImageAEOT is a new computational approach for modeling cell trajectories from independent datasets, combining an autoencoder to map images to a latent space with optimal transport to predict cell trajectories. Previous methods have used optimal transport for the analysis of single-cell RNA-seq data during differentiation [14], but the use of an autoencoder is crucial when working with images to define a common coordinate system of the data. While existing applications of machine learning in biological imaging are mainly focused on supervised classification tasks [18–20], ImageAEOT demonstrates the promise of machine learning for building predictive models and identifying relevant features in an unsupervised manner.

Recent studies have revealed a strong coupling between cellular mechanical state, chromatin packing and the biochemical interaction networks within a cell [40–45]. We therefore used nuclear images as a functional read-out to analyze cell-state transitions within the heterogeneous tissue microenvironment. In particular, we applied ImageAEOT to analyze the process of fibroblast activation and cancer progression, demonstrating that ImageAEOT can be used to identify the most salient image features of a particular cell state change without having access to continuous time data. Collectively, our results provide a quantitative framework to analyze cellular transitions at time scales relevant for developmental and disease processes, thereby opening novel routes for the identification of early disease biomarkers from imaging data.

## Supporting information

S1 Fig (a) Co-culture experiment: Representative maximum intensity projected images of control MCF7 cells, control NIH3T3 cells and MCF7-NIH3T3 co-culture cells in 3D collagen gel from Day 1 to Day 4. EpCAM (green) is enriched in MCF7 cells, Vimentin (red) is enriched in NIH3T3 cells; the nuclei are also labelled using DAPI (blue). Each image acquired is 1024 x 1024 pixels in size. To obtain a larger field of view, multiple images were stitched together to create a large image, consisting of 4 images (2 x 2) for Day 3 and Day 4 in the control MCF7 condition and 9 images (3x3) in the control NIH3T3 and co-culture conditions. Scale bar: 100μm. (b) Image processing workflow: The raw 3D images labelled for DNA using DAPI, acquired using a laser scanning confocal microscope, are filtered using a Gaussian blur and thresholded using an automated global thresholding method such as otsu to binarize the image and identify nuclear regions. Watershed is used to separate closeby nuclei. The resulting binary image is then used to identify individual nuclei as a 3D objects within a size range of 200-1300μm3. Each nucleus identified as a separate 3D object is visualized with distinct colors. In order to smoothen any irregular boundaries, a 3D convex hull is constructed and then the individual nuclei are cropped along their bounding rectangles and stored. From this set, the blurred out of focus nuclei or over-exposed nuclei are filtered out and then the remaining nuclei are used for further analysis.
(TIF)

S2 Fig (a) Architecture of variational autoencoder. The encoder used for mapping images to the latent space is shown on the left. This encoder takes images as input and returns Gaussian parameters in the latent space that correspond to this image. The decoder used for mapping from the latent space back into the image space is shown on the right. (b) VoxNet architecture used in the classification tasks. The input images are of size 32 × 32 × 32. The notation r × Conv3D-k (3 × 3 × 3) means that there are r 3D convolutional layers (one feeds into the other) each with k filters of size 3 × 3 × 3. MaxPool3D(2 × 2 × 2) indicates a 3D max pooling layer with pooling size 2 × 2 × 2. FC-k indicates a fully connected layer with k neurons. Note that the PReLU activation function is used in every convolutional layer while ReLU activation functions are used in the fully connected layers. Finally, batch normalization is followed by every convolutional layer.
(TIF)

S3 Fig (a-c) Training the variational autoencoder on co-culture NIH3T3 nuclei; 218 random images out of 4160 total are held-out for validation, and the remaining images are used to train the autoencoder. (a) Training and test loss curves of the variational autoencoder plotted over 1000 epochs. (b) Nuclear images generated from sampling random vectors in the latent space and mapping these to the image space. These random samples resemble nuclei, suggesting that the variational autoencoder learns the manifold of the image data. (c) Input and reconstructed images from Day 1 to Day 4 illustrating that the latent space captures the main visual features of the original images. (d-f) Hyperparameter tuning for the variational autoencoder over co-culture nuclei. (d-e) Training loss and test loss curves respectively for high, mid, or no regularization. (f, top row) Reconstruction results for each model. Models with no or mid-level regularization can reconstruct input images well, while models with high regularization do not. (f, bottom row) Sampling results for each model. Models with no regularization do not generate random samples as well as models with mid-level regularization, which suggests that the model with mid-level regularization best captures the manifold of nuclei images. (g-j) ImageAEOT applied to tracing trajectories of cancer cells in a co-culture system; 121 random images out of 2321 total are held-out for validation, and the remaining images are used to train the autoencoder. (g) Visualization of MCF7 nuclear images from Days 1-4 in both the image and latent space using an LDA plot. Note that the distributions of the data points in the LDA plot appear to coincide, suggesting that the MCF7 cells do not undergo drastic changes from Day 1 to 4. Day 1: black; Day 2: purple; Day 3: red; Day 4: green. (h) Predicted trajectories in the latent space using optimal transport. ImageAEOT was used to trace the trajectories of Day 1 MCF7 to Day 4 MCF7. Each black arrow is an example of a trajectory. (i) Visualization of the principal feature along the first linear discriminant. The nuclear images are of Day 1 MCF7 cells. The images below show the difference between the generated images along the first linear discriminant and the original image (blue: decrease in pixel intensity; red: increase in pixel intensity). These results suggest that MCF7 nuclei do not exhibit drastic changes other than a reduction of intensity. (j) Predicted trajectories mapped back to the image space. Note that only the first image in each sequence is a real Day 1 MCF7 nucleus; the remaining images are predicted and generated by ImageAEOT. Note that there are only small changes in the nuclei, other than a decrease in overall intensity.
(TIF)

S4 Fig (a-c) Pairwise classification of NIH3T3 cells from co-culture model in the latent space. (a) Classification results in the latent space using a linear model. Top: training and test loss curves for each pairwise comparison (Day 1, Day 2, Day 3, Day 4). Middle: training and test accuracy curves for each pairwise comparison. Bottom: Table of best training (red) and test (green) accuracy for each classification task. (b) Same as (a) but using a two-layer feedforward neural network. (c) Training and validation dataset sizes for each of the classification tasks. (d-f) Pairwise classification of NIH3T3 cells from co-culture model using VoxNet. (d) Accuracy and (e) loss curves for each pairwise comparison (Day 1, Day 2, Day 3, Day 4). (f) Table of best training (red) and test (green) accuracy for each classification task. 4-way classification results for co-culture NIH3T3 nuclei. Training and test loss curves for 4-way classification task (Day 1, Day 2, Day 3, Day 4) of co-culture NIH3T3 nuclei in the latent space using a linear model (g) and 2-layer feedforward neural network (h). (i-j) Confusion matrices for the classification tasks in (g-h). Each entry (X/Y) in row “A” and column “B” indicates that X nuclei of class “A” were classified as “B” in the training set and Y nuclei of class “A” were classified as “B” in the test set. (k) Same as (h,j) but for the 4-way classification task in the original image space using a deep convolutional neural network.
(TIF)

S5 Fig (a-c) Pairwise classification of MCF7 cells from co-culture model. (a) Classification results in the latent space using a linear model. Top: training and test loss curves for each pairwise comparison (Day 1, Day 2, Day 3, Day 4). Middle: training and test accuracy curves for each pairwise comparison. Bottom: Table of best training (red) and test (green) accuracy for each classification task. (b) Same as (a) but using a two-layer feedforward neural network. (c) Training and validation dataset sizes for each of the classification tasks. (d-f) Pairwise classification of MCF7 cells from co-culture model using VoxNet. (d) Accuracy and (e) loss curves for each pairwise comparison (Day 1, Day 2, Day 3, Day 4). (f) Table of best training (red) and test (green) accuracy for each classification task. 4-way classification results for co-culture MCF7 nuclei. (g-k) Training and test loss curves for 4-way classification task (Day 1, Day 2, Day 3, Day 4) of co-culture MCF7 nuclei using a linear model (g) and 2-layer feedforward neural network (h). (i-j) Confusion matrices for the classification tasks in (g-h). Each entry (X/Y) in row “A” and column “B” indicates that X nuclei of class “A” were classified as “B” in the training set and Y nuclei of class “A” were classified as “B” in the test set. (k) Same as (h,j) but for the 4-way classification task in the original image space using a deep convolutional neural network.
(TIF)

S6 Fig Feature ablation tables for co-culture nuclei. (a) Feature ablation table for logistic regression on NIH3T3 co-cultured cells. (b) Feature ablation table for logistic regression on MCF7 co-cultured cells.
(TIF)

S7 Fig (a-c) Training the variational autoencoder on various breast cell lines; 64 random images out of 1220 total are held-out for validation, and the remaining images are used to train the autoencoder (a) Training and test loss curves of variational autoencoder plotted over 1000 epochs. (b) Nuclear images generated from sampling random vectors in the latent space and mapped back to the image space. These random samples resemble real nuclei, suggesting that the variational autoencoder learns the image manifold. (c) Input and reconstructed images from different cell lines, illustrating that the latent space captures the main visual feature of the original images. (d-f) Hyperparameter tuning for variational autoencoder on breast cell lines. (d-e) Training loss and test loss curves respectively with high, mid, and no regularization. (f, top row) Reconstruction results for each model. Models with no or mid-level regularization can reconstruct input images well, while models with high regularization do not. (f, bottom row) Sampling results for each model. Models with no regularization do not generate random samples as well as models with mid-level regularization, which suggests that the model with mid-level regularization best captures the manifold of nuclear images.
(TIF)

S8 Fig (a-b) Pairwise classification of nuclei from breast cell lines in the latent space. (a) Classification results in the latent space using a linear model. Top: training and test loss curves for each pairwise comparison (HME1, MCF10A, MCF7, MDA-MB231). Middle: training and test accuracy curves for each pairwise comparison. Bottom: Table of best training (red) and test (green) accuracy for each classification task. (b) Same as (a) but using a two-layer feedforward neural network. For all tasks, the sizes of the training and validation datasets were 550 and 60 respectively. (c-e) Pairwise classification of nuclei from breast cell lines using VoxNet. (c) Accuracy and (d) loss curves for each pairwise comparison (HME1, MCF10A, MCF7, MDA-MB231). (e) Table of best training (red) and test (green) accuracy for each classification task. (f-k) 4-way classification results for nuclei from breast cell lines. Training and test loss curves for 4-way classification task of HME-1, MCF10A, MCF7 and MDA-MB231 nuclei using a linear model (f) and 2-layer feedforward neural network (g). (h-i) Confusion matrices for the classification tasks in (f-g). Each entry (X/Y) in row “A” and column “B” indicates that X nuclei of class “A” were classified as “B” in the training set and Y nuclei of class “A” were classified as “B” in the test set. (j-k) Same as (g,i) but for the 4-way classification task in the original image space using a deep convolutional neural network.
(TIF)

S9 Fig Principal features of change between cell lines. (a) Left: real MCF10A nuclear images. Right: heatmap of changes in pixel intensity of MCF10A nuclei after modulation along the first linear discriminant towards HME-1 nuclei. (b) Left: real MCF7 nuclear images. Right: heatmap of changes in pixel intensity of MFC-7 nuclei after modulation along the first linear discriminant towards MDA-MB231 nuclei. (c) Left: real HME-1 nuclear images. Right: heatmap of changes in pixel intensity of HME-1 nuclei after modulation along the first linear discriminant towards MCF10A nuclei. (d) Left: real MDA-MB231 nuclei images. Right: heatmap of changes in pixel intensity of MDA-MB231 nuclei after modulation along the first linear discriminant towards MCF7 nuclei. (e) Logistic regression and feature ablation table on the 4 breast cell lines.
(TIF)

S10 Fig Hyperparameter tuning for variational autoencoder applied to human breast tissues; 42 random images out of 798 total are held-out for validation, and the remaining images are used to train the autoencoder (a-b) Training loss and test loss curves respectively with high, mid, and no regularization. (c, top row) Reconstruction results for each model. Models with no or mid-level regularization can reconstruct input images well, while models with high regularization do not. (c, bottom row) Sampling results for each model. Models with no regularization do not generate random samples as well as models with mid-level regularization, suggesting that the model with mid-level regularization best captures the image manifold.
(TIF)

S11 Fig Comparison of ImageAEOT using a standard autoencoder (AE+OT) and an augmented autoencoder (augAE+OT) against Waddington-OT (OT, PCA+OT) on the proposed benchmark task. The evaluation metric shown is the area under the receiver operating characteristic curve (ROC-AUC) of the inferred probabilistic labels (score between 0 and 1, higher is better). ImageAEOT achieves considerable quantitative gains over the baseline. Morphology (Ecc) and Morphology (Roundness) refer to eccentricity and roundness of the cell; these labels were obtained using the Mahotas package.
(TIF)

S12 Fig Distributions of a principal feature of the cells (i.e. the first linear discriminant shown in x-axis of Fig 2C and 2D) from Day 1-4 are shown in the four box plots, overlaying the predicted distributions of the cells between Days 1 and Day 4 using ImageAEOT. For the predicted distributions, the line separating pink and green is the median; the lines separating green from purple and red from purple denote respectively the first and third quartiles; the blue extends to 1.5 times the interquartile range. The box-plots of the observed experimental distributions are overlaid on top. Note that the distributions of the predicted trajectories coincide with the true distributions, even though only Day 1 and Day 4 NIH3T3 nuclei were used to trace the trajectories.
(TIF)

## References

[1] Burgess DJ. Tracing cell-lineage histories. Nature Reviews Genetics. 2018;19(6):327 10.1038/s41576-018-0015-0 29713013

[2] Woodworth MB, Girskis KM, Walsh CA. Building a lineage from single cells: genetic techniques for cell lineage tracking. Nature Reviews Genetics. 2017;18(4):230 10.1038/nrg.2016.159 28111472 PMC5459401

[3] Kretzschmar K, Watt FM. Lineage tracing. Cell. 2012;148(1-2):33–45. 10.1016/j.cell.2012.01.002 22265400

[4] Trapnell C, Cacchiarelli D, Grimsby J, Pokharel P, Li S, Morse M, et al The dynamics and regulators of cell fate decisions are revealed by pseudotemporal ordering of single cells. Nature biotechnology. 2014;32(4):381 10.1038/nbt.2859 24658644 PMC4122333

[5] Qiu X, Mao Q, Tang Y, Wang L, Chawla R, Pliner HA, et al Reversed graph embedding resolves complex single-cell trajectories. Nature methods. 2017;14(10):979 10.1038/nmeth.4402 28825705 PMC5764547

[6] Guo M, Bao EL, Wagner M, Whitsett JA, Xu Y. SLICE: determining cell differentiation and lineage based on single cell entropy. Nucleic acids research. 2017;45(7):e54–e54. 10.1093/nar/gkw1278 27998929 PMC5397210

[7] Shin J, Berg DA, Zhu Y, Shin JY, Song J, Bonaguidi MA, et al Single-cell RNA-seq with waterfall reveals molecular cascades underlying adult neurogenesis. Cell stem cell. 2015;17(3):360–372. 10.1016/j.stem.2015.07.013 26299571 PMC8638014

[8] Ji Z, Ji H. TSCAN: Pseudo-time reconstruction and evaluation in single-cell RNA-seq analysis. Nucleic acids research. 2016;44(13):e117–e117. 10.1093/nar/gkw430 27179027 PMC4994863

[9] Marco E, Karp RL, Guo G, Robson P, Hart AH, Trippa L, et al Bifurcation analysis of single-cell gene expression data reveals epigenetic landscape. Proceedings of the National Academy of Sciences. 2014;111(52):E5643–E5650. 10.1073/pnas.1408993111 PMC4284553 25512504

[10] Bendall SC, Davis KL, Amir EaD, Tadmor MD, Simonds EF, Chen TJ, et al Single-cell trajectory detection uncovers progression and regulatory coordination in human B cell development. Cell. 2014;157(3):714–725. 10.1016/j.cell.2014.04.005 24766814 PMC4045247

[11] Setty M, Tadmor MD, Reich-Zeliger S, Angel O, Salame TM, Kathail P, et al Wishbone identifies bifurcating developmental trajectories from single-cell data. Nature biotechnology. 2016;34(6):637 10.1038/nbt.3569 27136076 PMC4900897

[12] Wolf FA, Hamey FK, Plass M, Solana J, Dahlin JS, Göttgens B, et al PAGA: graph abstraction reconciles clustering with trajectory inference through a topology preserving map of single cells. Genome biology. 2019;20(1):59 10.1186/s13059-019-1663-x 30890159 PMC6425583

[13] Weinreb C, Wolock S, Tusi BK, Socolovsky M, Klein AM. Fundamental limits on dynamic inference from single-cell snapshots. Proceedings of the National Academy of Sciences. 2018;115(10):E2467–E2476. 10.1073/pnas.1714723115 PMC5878004 29463712

[14] Schiebinger G, Shu J, Tabaka M, Cleary B, Subramanian V, Solomon A, et al Reconstruction of developmental landscapes by optimal-transport analysis of single-cell gene expression sheds light on cellular reprogramming. Cell. 2019;176(4):928–943.e22. 30712874 10.1016/j.cell.2019.01.006 PMC6402800

[15] LeCun Y, Bengio Y, Hinton G. Deep learning. Nature. 2015;521(7553):436 10.1038/nature14539 26017442

[16] Goodfellow I, Bengio Y, Courville A. Deep Learning. MIT press; 2016.

[17] Bengio Y, et al Learning deep architectures for AI. Foundations and Trends^®^ in Machine Learning. 2009;2(1):1–127. 10.1561/2200000006

[18] Sommer C, Gerlich DW. Machine learning in cell biology–teaching computers to recognize phenotypes. J Cell Sci. 2013;126(24):5529–5539. 10.1242/jcs.123604 24259662

[19] Shen D, Wu G, Suk HI. Deep learning in medical image analysis. Annual Review of Biomedical Engineering. 2017;19:221–248. 10.1146/annurev-bioeng-071516-044442 28301734 PMC5479722

[20] Grys BT, Lo DS, Sahin N, Kraus OZ, Morris Q, Boone C, et al Machine learning and computer vision approaches for phenotypic profiling. J Cell Biol. 2017;216(1):65–71. 10.1083/jcb.201610026 27940887 PMC5223612

[21] Hinton GE, Salakhutdinov RR. Reducing the dimensionality of data with neural networks. Science. 2006;313(5786):504–507. 10.1126/science.1127647 16873662

[22] Hinton GE, Zemel RS. Autoencoders, minimum description length and Helmholtz free energy In: Advances in Neural Information Processing Systems; 1994 p. 3–10.

[23] Kingma DP, Welling M. Auto-encoding variational bayes. arXiv preprint arXiv:13126114. 2013;.

[24] Monge G. Mémoire sur la théorie des déblais et des remblais. Histoire de l’Académie Royale des Sciences de Paris. 1781;.

[25] Kantorovich LV. On the translocation of masses. In: Dokl. Akad. Nauk. USSR (NS). vol. 37; 1942. p. 199–201.

[26] Villani C. Optimal Transport: Old and New. vol. 338 Springer Science & Business Media; 2008.

[27] Cuturi M. Sinkhorn distances: Lightspeed computation of optimal transport In: Advances in Neural Information Processing Systems; 2013 p. 2292–2300.

[28] Makhzani A, Shlens J, Jaitly N, Goodfellow I, Frey B. Adversarial autoencoders. arXiv preprint arXiv:151105644. 2015;.

[29] Tolstikhin I, Bousquet O, Gelly S, Schoelkopf B. Wasserstein auto-encoders. arXiv preprint arXiv:171101558. 2017;.

[30] Weinreb C, Rodriguez-Fraticelli AE, Camargo FD, Klein AM. Lineage tracing on transcriptional landscapes links state to fate during differentiation. bioRxiv. 2018; p. 467886. 10.1126/science.aaw3381 PMC7608074 31974159

[31] Kalluri R. The biology and function of fibroblasts in cancer. Nature Reviews Cancer. 2016;16(9):582 10.1038/nrc.2016.73 27550820

[32] Yamauchi M, Barker TH, Gibbons DL, Kurie JM. The fibrotic tumor stroma. Journal of Clinical Investigation. 2018;128(1):16–25. 10.1172/JCI93554 29293090 PMC5749516

[33] Franco OE, Shaw AK, Strand DW, Hayward SW. Cancer associated fibroblasts in cancer pathogenesis In: Seminars in Cell & Developmental Biology. vol. 21 Elsevier; 2010 p. 33–39. 19896548 10.1016/j.semcdb.2009.10.010 PMC2823834

[34] Coelho LP. Mahotas: Open source software for scriptable computer vision. arXiv preprint arXiv:12114907. 2012;.

[35] Venkatachalapathy S, Jokhun DS, Shivashankar GV. Multivariate analysis reveals activation-primed fibroblast geometric states in engineered 3D tumor microenvironments. Molecular Biology of the Cell. 2020;. 32023167 10.1091/mbc.E19-08-0420 PMC7185960

[36] Uhler C, Shivashankar G. Nuclear mechanopathology and cancer diagnosis. Trends in Cancer. 2018;4(4):320–331. 10.1016/j.trecan.2018.02.009 29606315

[37] Griffiths JA, Scialdone A, Marioni JC. Using single-cell genomics to understand developmental processes and cell fate decisions. Molecular Systems Biology. 2018;14(4):e8046 10.15252/msb.20178046 29661792 PMC5900446

[38] Schwartzman O, Tanay A. Single-cell epigenomics: Techniques and emerging applications. Nature Reviews Genetics. 2015;16(12):716 10.1038/nrg3980 26460349

[39] Su Y, Shi Q, Wei W. Single cell proteomics in biomedicine: High-dimensional data acquisition, visualization, and analysis. Proteomics. 2017;17(3-4):1600267 10.1002/pmic.201600267 PMC5554115 28128880

[40] Uhler C, Shivashankar G. Regulation of genome organization and gene expression by nuclear mechanotransduction. Nature Reviews Molecular Cell Biology. 2017;18(12):717 10.1038/nrm.2017.101 29044247

[41] Belyaeva A, Venkatachalapathy S, Nagarajan M, Shivashankar G, Uhler C. Network analysis identifies chromosome intermingling regions as regulatory hotspots for transcription. Proceedings of the National Academy of Sciences. 2017;114(52):13714–13719. 10.1073/pnas.1708028115 PMC5748172 29229825

[42] Lanctôt C, Cheutin T, Cremer M, Cavalli G, Cremer T. Dynamic genome architecture in the nuclear space: regulation of gene expression in three dimensions. Nature Reviews Genetics. 2007;8(2):104 10.1038/nrg2041 17230197

[43] Bustin M, Misteli T. Nongenetic functions of the genome. Science. 2016;352(6286):aad6933 10.1126/science.aad6933 27151873 PMC6312727

[44] Kirby TJ, Lammerding J. Emerging views of the nucleus as a cellular mechanosensor. Nature Cell Biology. 2018; p. 1. 29467443 10.1038/s41556-018-0038-y PMC6440800

[45] Cho S, Irianto J, Discher DE. Mechanosensing by the nucleus: From pathways to scaling relationships. J Cell Biol. 2017;216(2):305–315. 10.1083/jcb.201610042 28043971 PMC5294790

[46] Pedregosa F, Varoquaux G, Gramfort A, Michel V, Thirion B, Grisel O, et al Scikit-learn: Machine Learning in Python. Journal of Machine Learning Research. 2011;12:2825–2830.

[47] Paszke A, Gross S, Chintala S, Chanan G, Yang E, DeVito Z, et al. Automatic differentiation in pytorch. https://openreviewnet/pdf?id=BJJsrmfCZ. 2017;.

[48] Kingma DP, Ba J. Adam: A method for stochastic optimization. arXiv preprint arXiv:14126980. 2014;.

[49] Maturana D, Scherer S. Voxnet: A 3d convolutional neural network for real-time object recognition. In: 2015 IEEE/RSJ International Conference on Intelligent Robots and Systems (IROS); 2015. p. 922–928.

[50] Makhija E, Jokhun DS, Shivashankar GV. Nuclear deformability and telomere dynamics are regulated by cell geometric constraints. Proceedings of the National Academy of Sciences, USA. 2016;113(1):E32–E40. 10.1073/pnas.1513189113 PMC4711833 26699462

[51] Schindelin J, Arganda-Carreras I, Frise E, Kaynig V, Longair M, Pietzsch T, et al Fiji: an open-source platform for biological-image analysis. Nature Methods. 2012;9(7):676 10.1038/nmeth.2019 22743772 PMC3855844

