# Supplementary figures and images for: Predicting cell lineages using autoencoders and optimal transport
Source: PLoS Comput Biol. 2020 Apr 28;16(4):e1007828. doi: 10.1371/journal.pcbi.1007828 (PMC7209334; doi:10.1371/journal.pcbi.1007828)

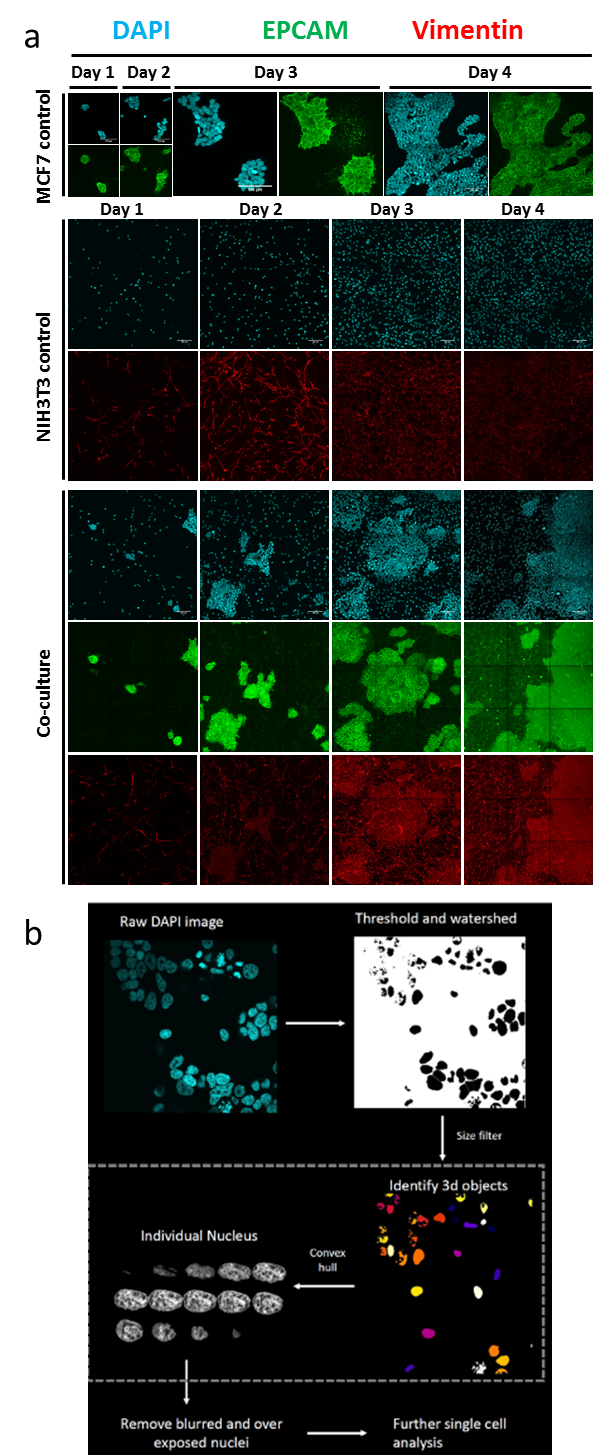

Supplement: S1 Fig — (a) Co-culture experiment: Representative maximum intensity projected images of control MCF7 cells, control NIH3T3 cells and MCF7-NIH3T3 co-culture cells in 3D collagen gel from Day 1 to Day 4. EpCAM (green) is enriched in MCF7 cells, Vimentin (red) is enriched in NIH3T3 cells; the nuclei are also labelled using DAPI (blue). Each image acquired is 1024 x 1024 pixels in size. To obtain a larger field of view, multiple images were stitched together to create a large image, consisting of 4 images (2 x 2) for Day 3 and Day 4 in the control MCF7 condition and 9 images (3x3) in the control NIH3T3 and co-culture conditions. Scale bar: 100μm. (b) Image processing workflow: The raw 3D images labelled for DNA using DAPI, acquired using a laser scanning confocal microscope, are filtered using a Gaussian blur and thresholded using an automated global thresholding method such as otsu to binarize the image and identify nuclear regions. Watershed is used to separate closeby nuclei. The resulting binary image is then used to identify individual nuclei as a 3D objects within a size range of 200-1300μm3. Each nucleus identified as a separate 3D object is visualized with distinct colors. In order to smoothen any irregular boundaries, a 3D convex hull is constructed and then the individual nuclei are cropped along their bounding rectangles and stored. From this set, the blurred out of focus nuclei or over-exposed nuclei are filtered out and then the remaining nuclei are used for further analysis. (TIF) [file pcbi.1007828.s001.tif]

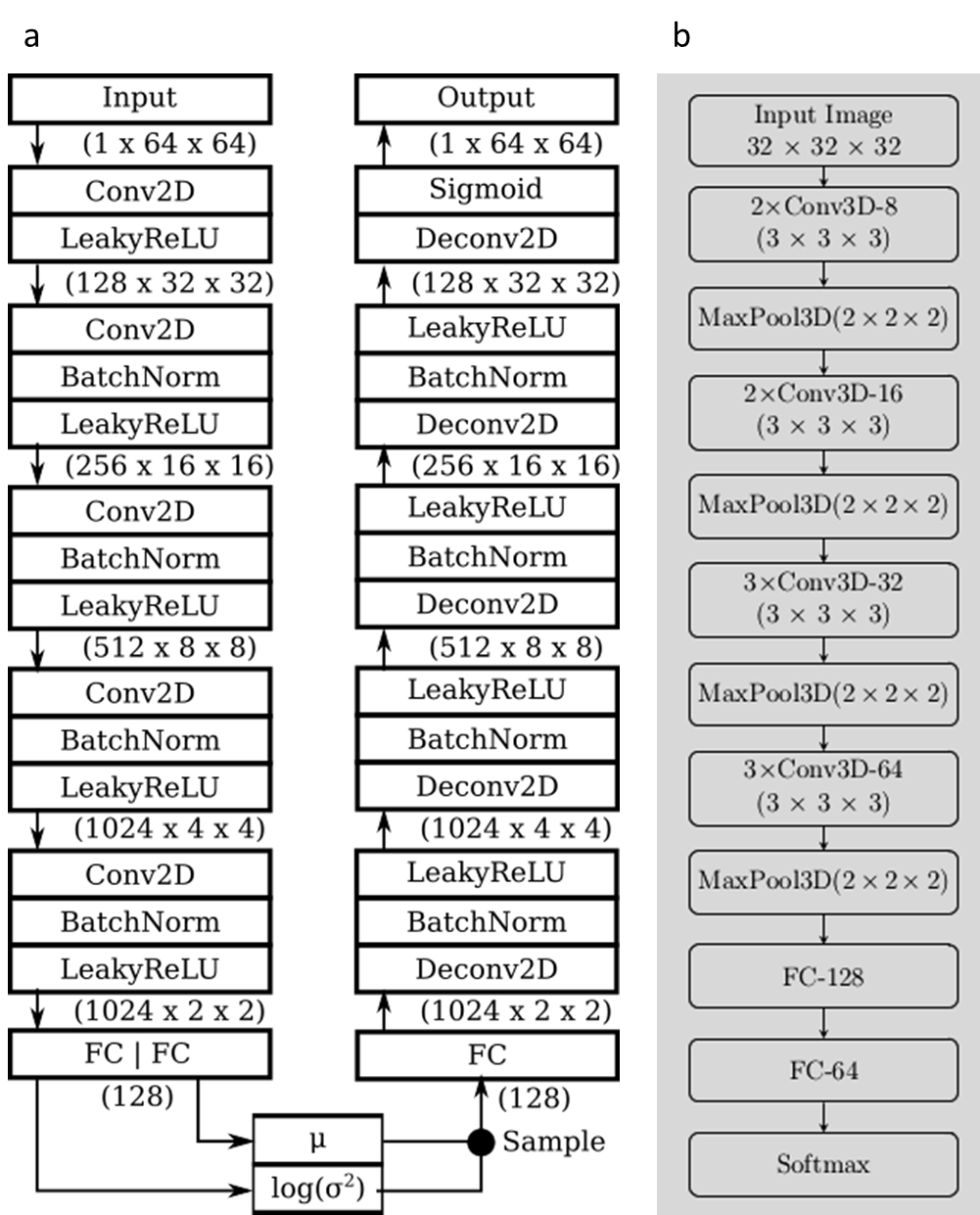

Supplement: S2 Fig — (a) Architecture of variational autoencoder. The encoder used for mapping images to the latent space is shown on the left. This encoder takes images as input and returns Gaussian parameters in the latent space that correspond to this image. The decoder used for mapping from the latent space back into the image space is shown on the right. (b) VoxNet architecture used in the classification tasks. The input images are of size 32 × 32 × 32. The notation r × Conv3D-k (3 × 3 × 3) means that there are r 3D convolutional layers (one feeds into the other) each with k filters of size 3 × 3 × 3. MaxPool3D(2 × 2 × 2) indicates a 3D max pooling layer with pooling size 2 × 2 × 2. FC-k indicates a fully connected layer with k neurons. Note that the PReLU activation function is used in every convolutional layer while ReLU activation functions are used in the fully connected layers. Finally, batch normalization is followed by every convolutional layer. (TIF) [file pcbi.1007828.s002.tif]

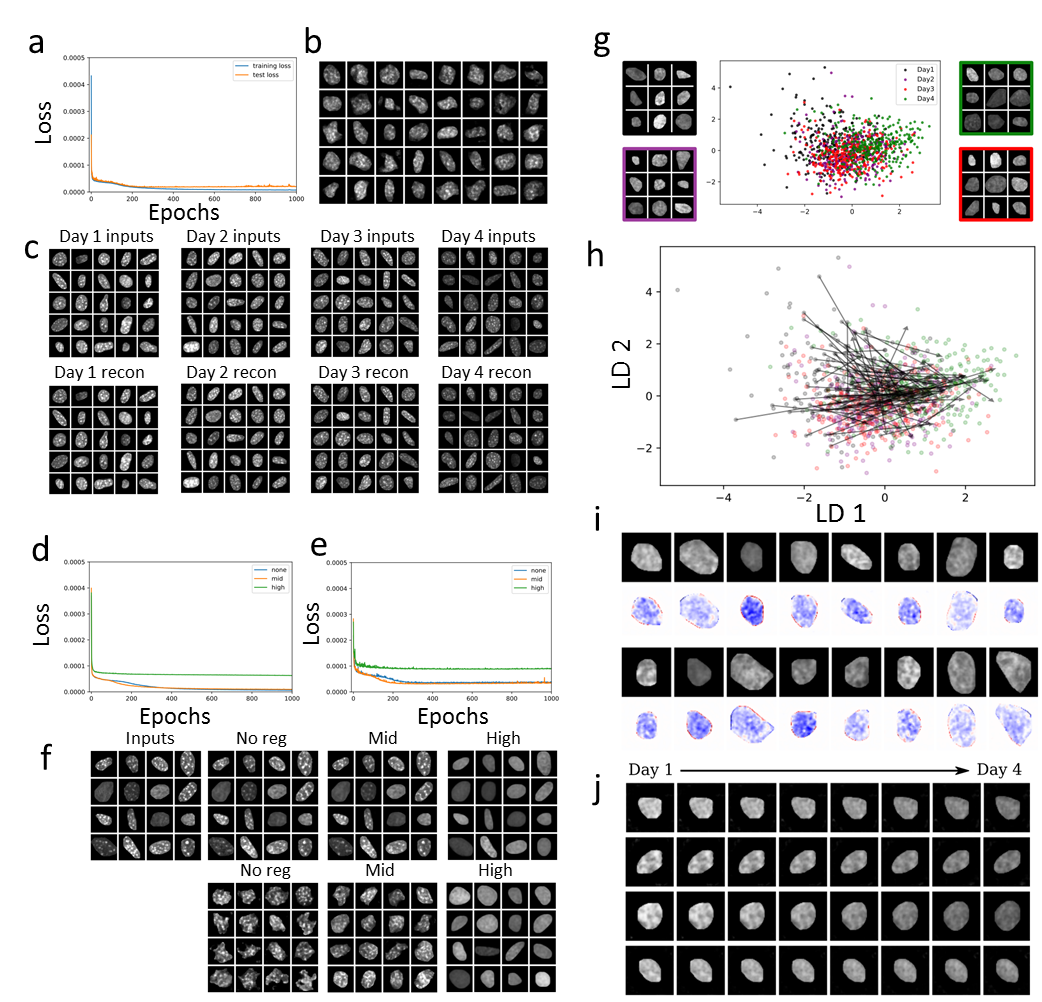

Supplement: S3 Fig — (a) Training and test loss curves of the variational autoencoder plotted over 1000 epochs. (b) Nuclear images generated from sampling random vectors in the latent space and mapping these to the image space. These random samples resemble nuclei, suggesting that the variational autoencoder learns the manifold of the image data. (c) Input and reconstructed images from Day 1 to Day 4 illustrating that the latent space captures the main visual features of the original images. (d-f) Hyperparameter tuning for the variational autoencoder over co-culture nuclei. (d-e) Training loss and test loss curves respectively for high, mid, or no regularization. (f, top row) Reconstruction results for each model. Models with no or mid-level regularization can reconstruct input images well, while models with high regularization do not. (f, bottom row) Sampling results for each model. Models with no regularization do not generate random samples as well as models with mid-level regularization, which suggests that the model with mid-level regularization best captures the manifold of nuclei images. (g-j) ImageAEOT applied to tracing trajectories of cancer cells in a co-culture system; 121 random images out of 2321 total are held-out for validation, and the remaining images are used to train the autoencoder. (g) Visualization of MCF7 nuclear images from Days 1-4 in both the image and latent space using an LDA plot. Note that the distributions of the data points in the LDA plot appear to coincide, suggesting that the MCF7 cells do not undergo drastic changes from Day 1 to 4. Day 1: black; Day 2: purple; Day 3: red; Day 4: green. (h) Predicted trajectories in the latent space using optimal transport. ImageAEOT was used to trace the trajectories of Day 1 MCF7 to Day 4 MCF7. Each black arrow is an example of a trajectory. (i) Visualization of the principal feature along the first linear discriminant. The nuclear images are of Day 1 MCF7 cells. The images below show the difference between the gen [file pcbi.1007828.s003.tif]

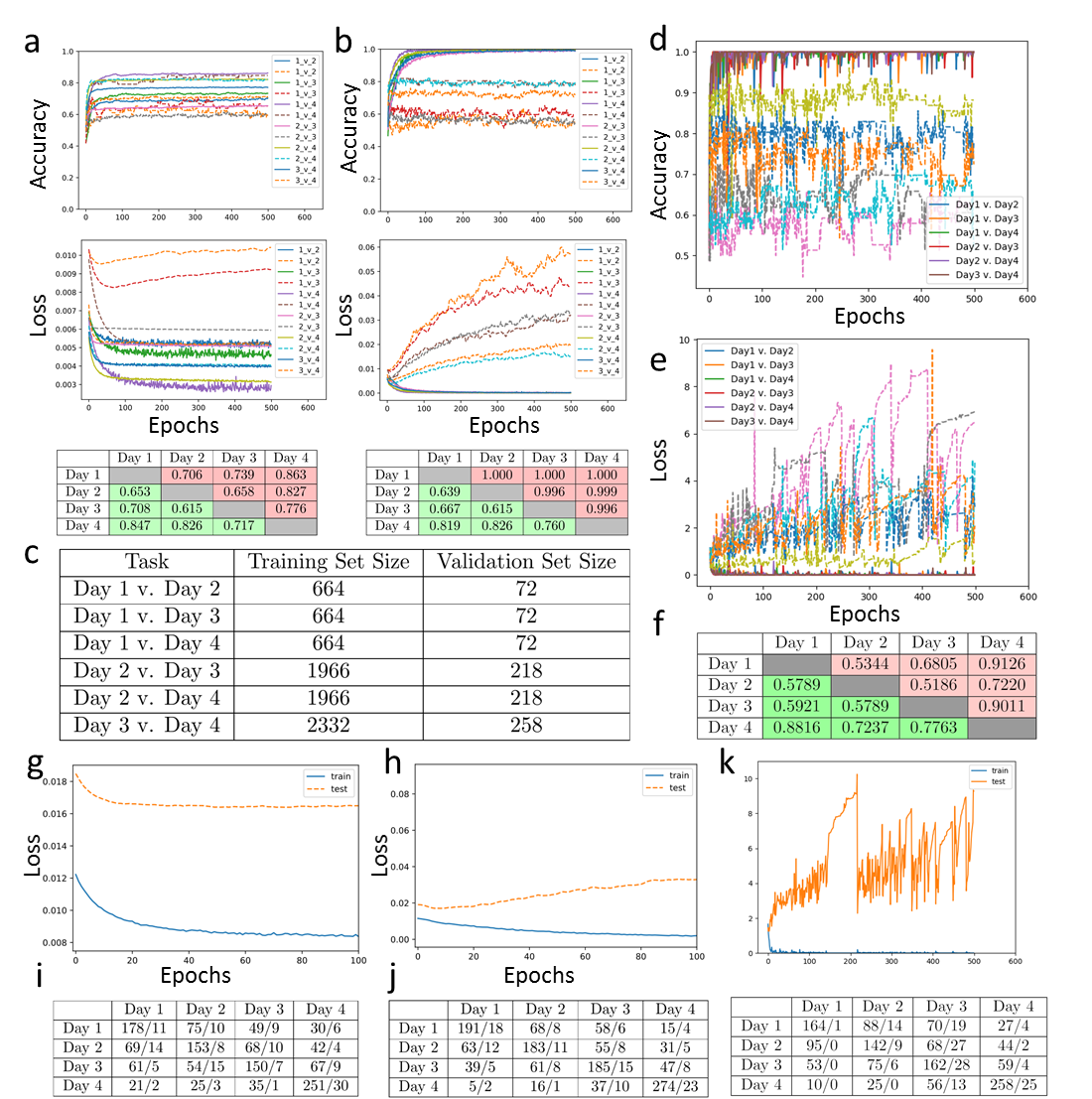

Supplement: S4 Fig — (a) Classification results in the latent space using a linear model. Top: training and test loss curves for each pairwise comparison (Day 1, Day 2, Day 3, Day 4). Middle: training and test accuracy curves for each pairwise comparison. Bottom: Table of best training (red) and test (green) accuracy for each classification task. (b) Same as (a) but using a two-layer feedforward neural network. (c) Training and validation dataset sizes for each of the classification tasks. (d-f) Pairwise classification of NIH3T3 cells from co-culture model using VoxNet. (d) Accuracy and (e) loss curves for each pairwise comparison (Day 1, Day 2, Day 3, Day 4). (f) Table of best training (red) and test (green) accuracy for each classification task. 4-way classification results for co-culture NIH3T3 nuclei. Training and test loss curves for 4-way classification task (Day 1, Day 2, Day 3, Day 4) of co-culture NIH3T3 nuclei in the latent space using a linear model (g) and 2-layer feedforward neural network (h). (i-j) Confusion matrices for the classification tasks in (g-h). Each entry (X/Y) in row “A” and column “B” indicates that X nuclei of class “A” were classified as “B” in the training set and Y nuclei of class “A” were classified as “B” in the test set. (k) Same as (h,j) but for the 4-way classification task in the original image space using a deep convolutional neural network. (TIF) [file pcbi.1007828.s004.tif]

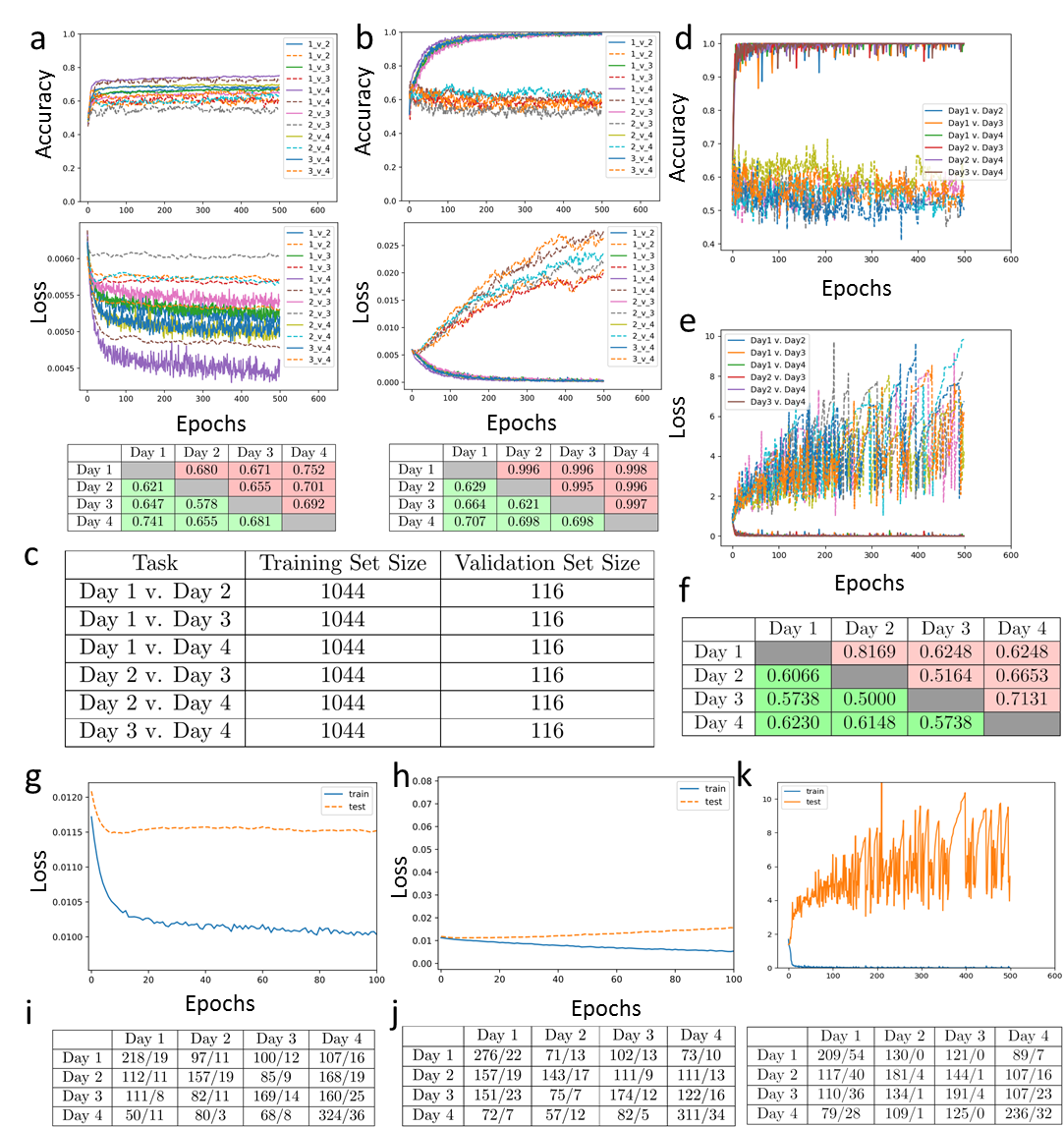

Supplement: S5 Fig — (a) Classification results in the latent space using a linear model. Top: training and test loss curves for each pairwise comparison (Day 1, Day 2, Day 3, Day 4). Middle: training and test accuracy curves for each pairwise comparison. Bottom: Table of best training (red) and test (green) accuracy for each classification task. (b) Same as (a) but using a two-layer feedforward neural network. (c) Training and validation dataset sizes for each of the classification tasks. (d-f) Pairwise classification of MCF7 cells from co-culture model using VoxNet. (d) Accuracy and (e) loss curves for each pairwise comparison (Day 1, Day 2, Day 3, Day 4). (f) Table of best training (red) and test (green) accuracy for each classification task. 4-way classification results for co-culture MCF7 nuclei. (g-k) Training and test loss curves for 4-way classification task (Day 1, Day 2, Day 3, Day 4) of co-culture MCF7 nuclei using a linear model (g) and 2-layer feedforward neural network (h). (i-j) Confusion matrices for the classification tasks in (g-h). Each entry (X/Y) in row “A” and column “B” indicates that X nuclei of class “A” were classified as “B” in the training set and Y nuclei of class “A” were classified as “B” in the test set. (k) Same as (h,j) but for the 4-way classification task in the original image space using a deep convolutional neural network. (TIF) [file pcbi.1007828.s005.tif]

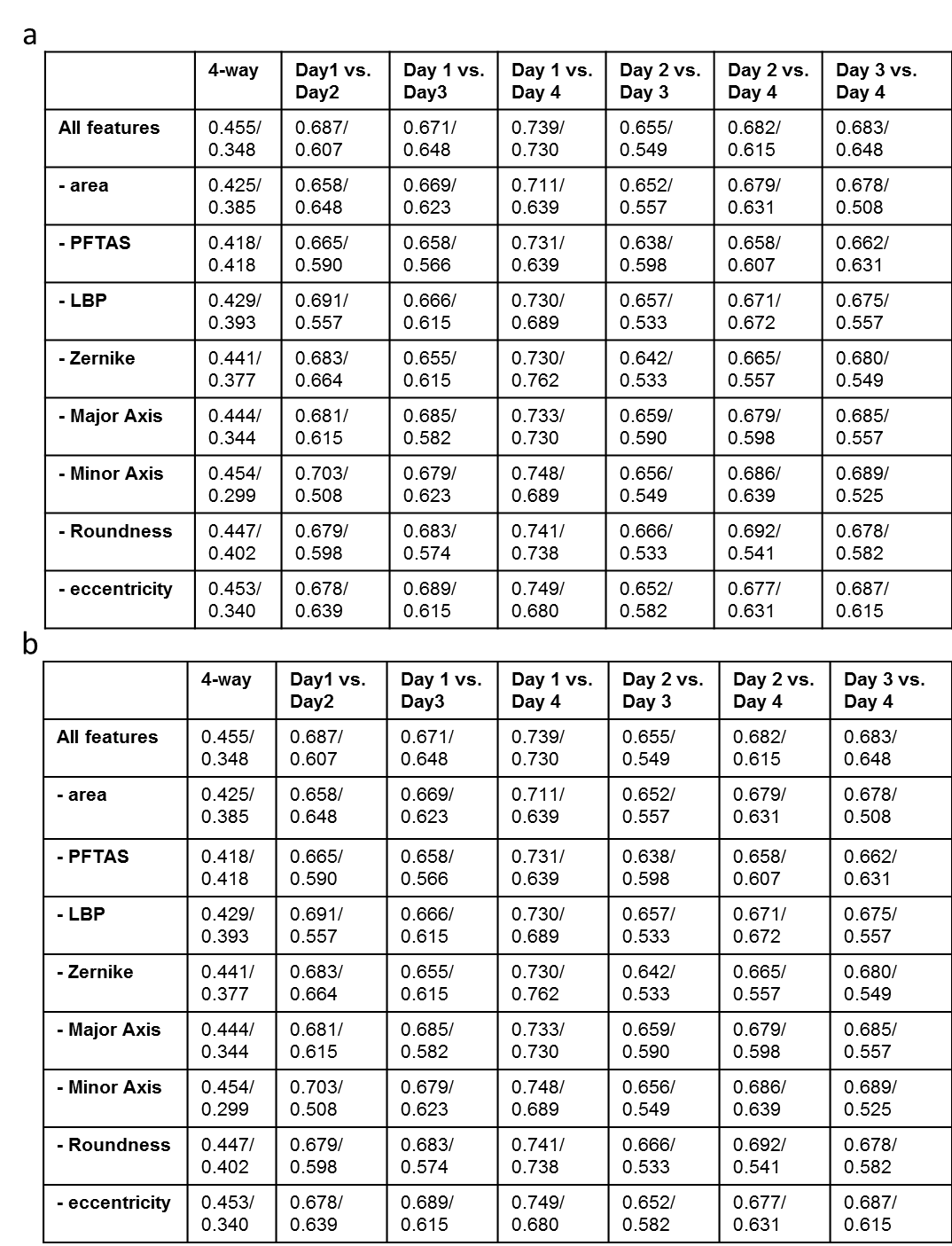

Supplement: S6 Fig — (a) Feature ablation table for logistic regression on NIH3T3 co-cultured cells. (b) Feature ablation table for logistic regression on MCF7 co-cultured cells. (TIF) [file pcbi.1007828.s006.tif]

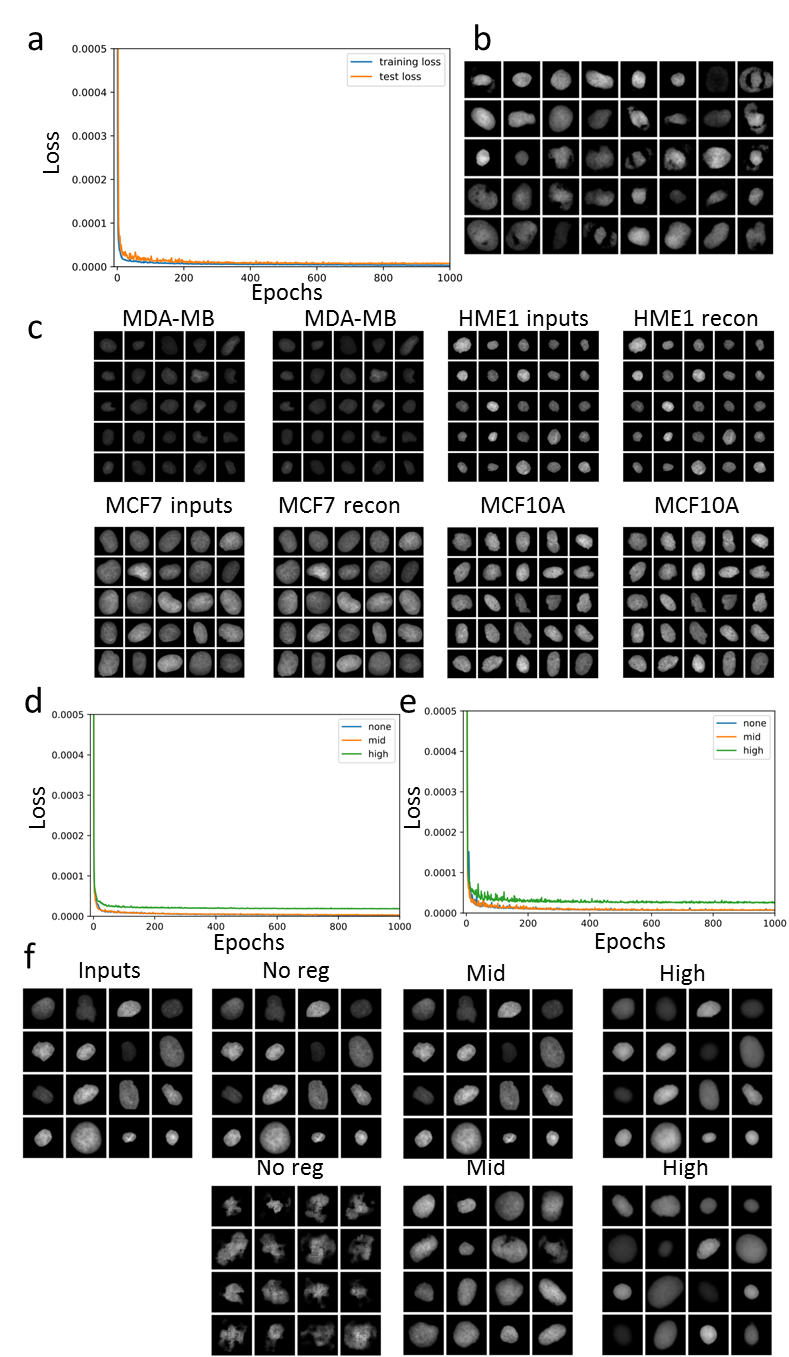

Supplement: S7 Fig — (a-c) Training the variational autoencoder on various breast cell lines; 64 random images out of 1220 total are held-out for validation, and the remaining images are used to train the autoencoder (a) Training and test loss curves of variational autoencoder plotted over 1000 epochs. (b) Nuclear images generated from sampling random vectors in the latent space and mapped back to the image space. These random samples resemble real nuclei, suggesting that the variational autoencoder learns the image manifold. (c) Input and reconstructed images from different cell lines, illustrating that the latent space captures the main visual feature of the original images. (d-f) Hyperparameter tuning for variational autoencoder on breast cell lines. (d-e) Training loss and test loss curves respectively with high, mid, and no regularization. (f, top row) Reconstruction results for each model. Models with no or mid-level regularization can reconstruct input images well, while models with high regularization do not. (f, bottom row) Sampling results for each model. Models with no regularization do not generate random samples as well as models with mid-level regularization, which suggests that the model with mid-level regularization best captures the manifold of nuclear images. (TIF) [file pcbi.1007828.s007.tif]

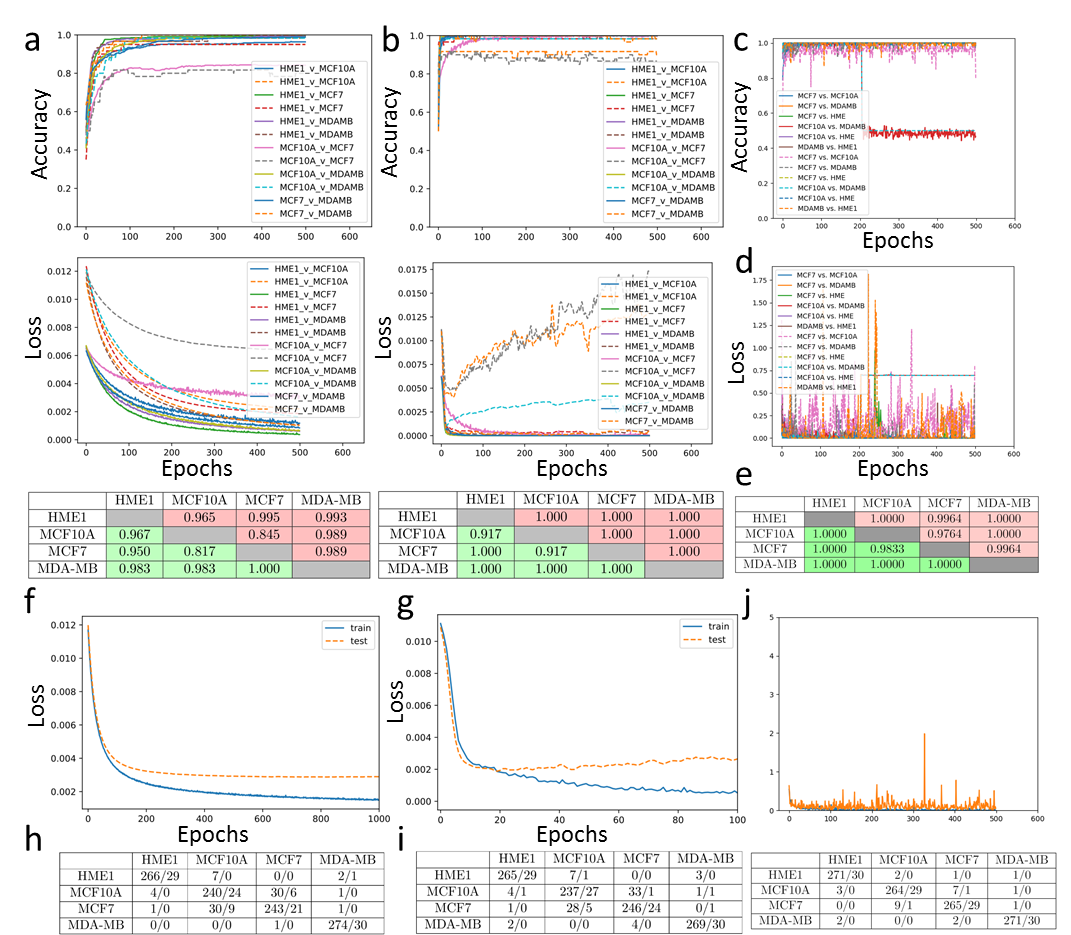

Supplement: S8 Fig — (a) Classification results in the latent space using a linear model. Top: training and test loss curves for each pairwise comparison (HME1, MCF10A, MCF7, MDA-MB231). Middle: training and test accuracy curves for each pairwise comparison. Bottom: Table of best training (red) and test (green) accuracy for each classification task. (b) Same as (a) but using a two-layer feedforward neural network. For all tasks, the sizes of the training and validation datasets were 550 and 60 respectively. (c-e) Pairwise classification of nuclei from breast cell lines using VoxNet. (c) Accuracy and (d) loss curves for each pairwise comparison (HME1, MCF10A, MCF7, MDA-MB231). (e) Table of best training (red) and test (green) accuracy for each classification task. (f-k) 4-way classification results for nuclei from breast cell lines. Training and test loss curves for 4-way classification task of HME-1, MCF10A, MCF7 and MDA-MB231 nuclei using a linear model (f) and 2-layer feedforward neural network (g). (h-i) Confusion matrices for the classification tasks in (f-g). Each entry (X/Y) in row “A” and column “B” indicates that X nuclei of class “A” were classified as “B” in the training set and Y nuclei of class “A” were classified as “B” in the test set. (j-k) Same as (g,i) but for the 4-way classification task in the original image space using a deep convolutional neural network. (TIF) [file pcbi.1007828.s008.tif]

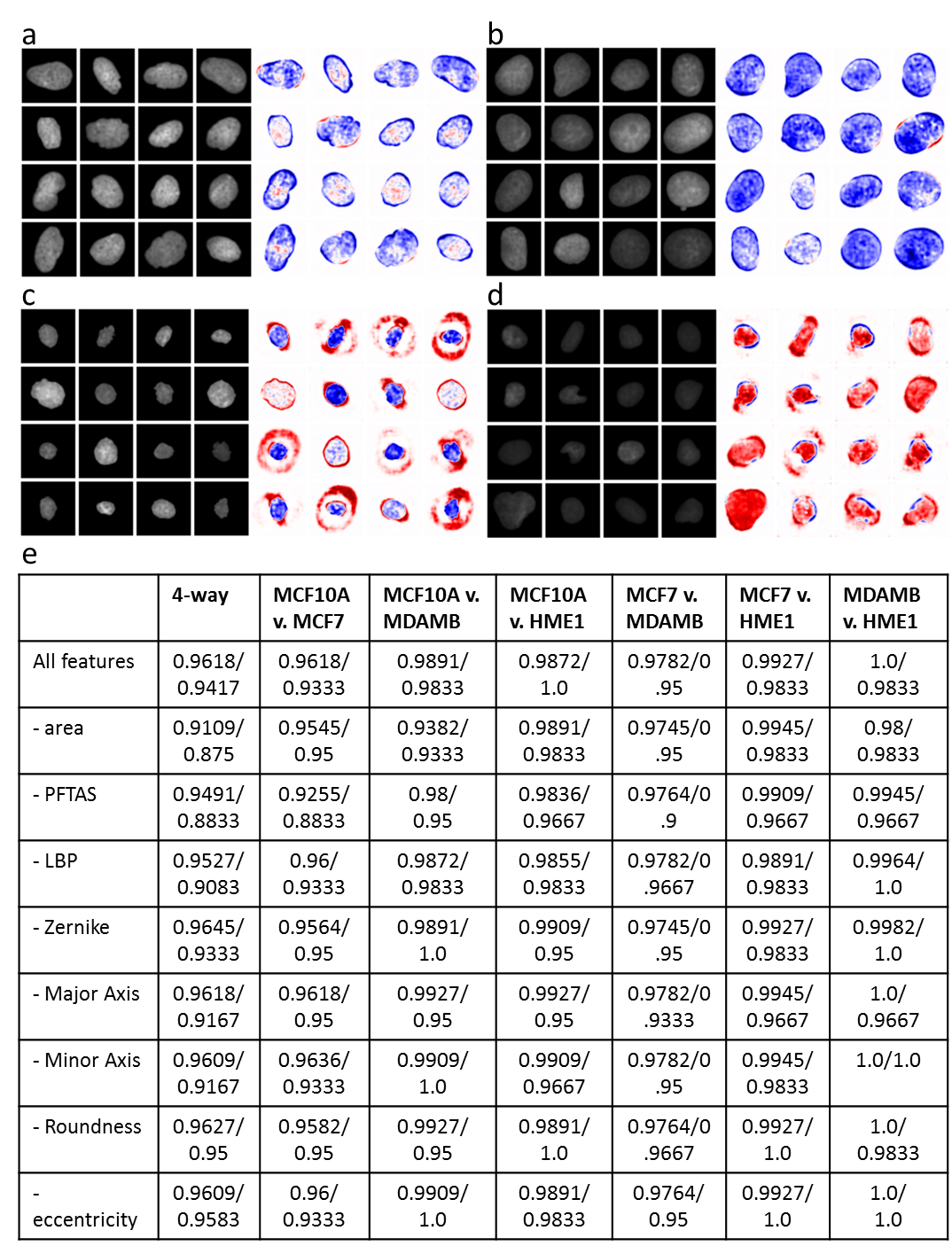

Supplement: S9 Fig — (a) Left: real MCF10A nuclear images. Right: heatmap of changes in pixel intensity of MCF10A nuclei after modulation along the first linear discriminant towards HME-1 nuclei. (b) Left: real MCF7 nuclear images. Right: heatmap of changes in pixel intensity of MFC-7 nuclei after modulation along the first linear discriminant towards MDA-MB231 nuclei. (c) Left: real HME-1 nuclear images. Right: heatmap of changes in pixel intensity of HME-1 nuclei after modulation along the first linear discriminant towards MCF10A nuclei. (d) Left: real MDA-MB231 nuclei images. Right: heatmap of changes in pixel intensity of MDA-MB231 nuclei after modulation along the first linear discriminant towards MCF7 nuclei. (e) Logistic regression and feature ablation table on the 4 breast cell lines. (TIF) [file pcbi.1007828.s009.tif]

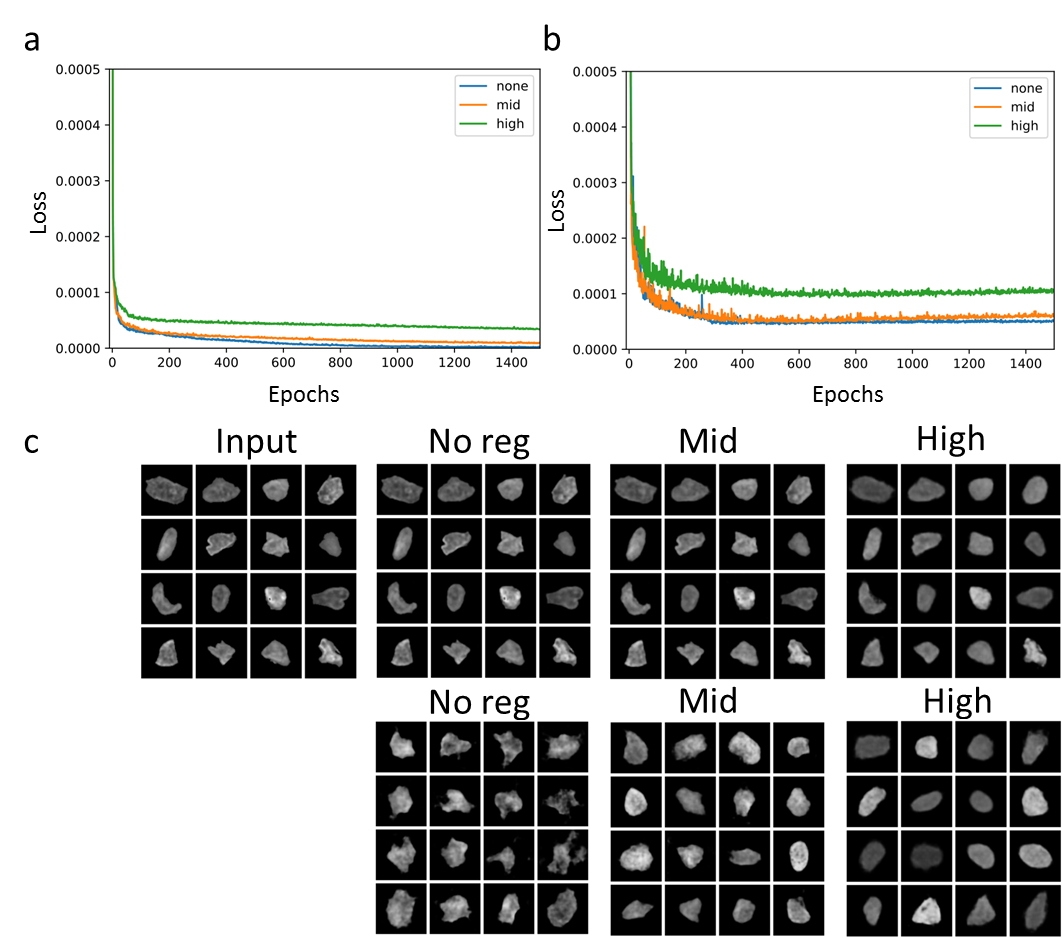

Supplement: S10 Fig — Hyperparameter tuning for variational autoencoder applied to human breast tissues; 42 random images out of 798 total are held-out for validation, and the remaining images are used to train the autoencoder (a-b) Training loss and test loss curves respectively with high, mid, and no regularization. (c, top row) Reconstruction results for each model. Models with no or mid-level regularization can reconstruct input images well, while models with high regularization do not. (c, bottom row) Sampling results for each model. Models with no regularization do not generate random samples as well as models with mid-level regularization, suggesting that the model with mid-level regularization best captures the image manifold. (TIF) [file pcbi.1007828.s010.tif]

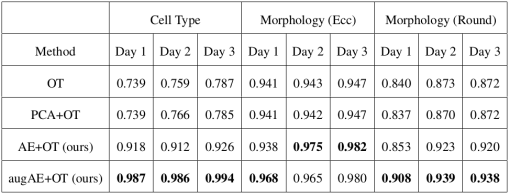

Supplement: S11 Fig — The evaluation metric shown is the area under the receiver operating characteristic curve (ROC-AUC) of the inferred probabilistic labels (score between 0 and 1, higher is better). ImageAEOT achieves considerable quantitative gains over the baseline. Morphology (Ecc) and Morphology (Roundness) refer to eccentricity and roundness of the cell; these labels were obtained using the Mahotas package. (TIF) [file pcbi.1007828.s011.tif]

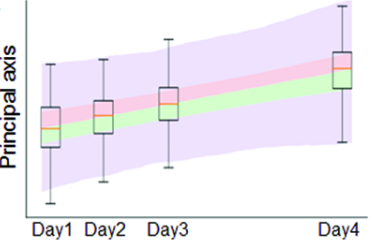

Supplement: S12 Fig — For the predicted distributions, the line separating pink and green is the median; the lines separating green from purple and red from purple denote respectively the first and third quartiles; the blue extends to 1.5 times the interquartile range. The box-plots of the observed experimental distributions are overlaid on top. Note that the distributions of the predicted trajectories coincide with the true distributions, even though only Day 1 and Day 4 NIH3T3 nuclei were used to trace the trajectories. (TIF) [file pcbi.1007828.s012.tif]
